# Holothurians have a reduced GPCR and odorant receptor-like repertoire compared to other echinoderms

**DOI:** 10.1038/s41598-020-60167-3

**Published:** 2020-02-25

**Authors:** Nathalie Marquet, João C. R. Cardoso, Bruno Louro, Stefan A. Fernandes, Sandra C. Silva, Adelino V. M. Canário

**Affiliations:** CCMAR - Centre of Marine Sciences, University of Algarve, Campus de Gambelas, 8005-139 Faro, Portugal

**Keywords:** Transcriptomics, Animal behaviour

## Abstract

Sea cucumbers lack vision and rely on chemical sensing to reproduce and survive. However, how they recognize and respond to environmental cues remains unknown. Possible candidates are the odorant receptors (ORs), a diverse family of G protein-coupled receptors (GPCRs) involved in olfaction. The present study aimed at characterizing the chemosensory GPCRs in sea cucumbers. At least 246 distinct GPCRs, of which *ca*. 20% putative ORs, were found in a transcriptome assembly of putative chemosensory (tentacles, oral cavity, calcareous ring, and papillae/tegument) and reproductive (ovary and testis) tissues from *Holothuria arguinensis* (57 ORs) and in the *Apostichopus japonicus* genome (79 ORs). The sea cucumber ORs clustered with those of sea urchin and starfish into four main clades of gene expansions sharing a common ancestor and evolving under purifying selection. However, the sea cucumber ORs repertoire was the smallest among the echinoderms and the olfactory receptor signature motif LxxPxYxxxxxLxxxDxxxxxxxxP was better conserved in cluster OR-l1 which also had more members. ORs were expressed in tentacles, oral cavity, calcareous ring, and papillae/tegument, supporting their potential role in chemosensing. This study is the first comprehensive survey of chemosensory GPCRs in sea cucumbers, and provides the molecular basis to understand how they communicate.

## Introduction

All living organisms perceive and respond to chemical cues in their environment, which mediate a variety of activities such as feeding, predator avoidance, mating and social behaviours^1,2^. These cues can be detected over long and short distances, and include a large diversity of molecules ranging from amino acids and nucleic acids, to small volatile compounds, peptides and proteins (see review^3^). To detect and discriminate chemical cues, animals have developed complex chemosensory organs, including the olfactory organs of vertebrates, which contain a large repertoire of chemosensory receptors^4^. Although well characterized in some animals^5–7^, chemosensory receptors remain largely undescribed in many metazoan lineages.

A large group of chemosensory receptors belong to the G protein-coupled receptors (GPCRs), one of the largest superfamilies of seven transmembrane domain receptors found in metazoans^8^. GPCRs convert extracellular stimuli, ranging from small molecules and photons to peptides and proteins, into intracellular biochemical signals via multiple signalling cascades (mostly cAMP and calcium secondary messengers)^9^. The large variety of ligands is reflected in the structural diversity of GPCRs which are classified into five main families based on their sequence similarity (GRAFS system): glutamate (G), rhodopsin (R), adhesion (A), frizzled (F) and secretin (S)^10^. Chemosensory functions have been associated with the glutamate-receptor family and the rhodopsin-type family^11,12^. The latter contains the largest number and the most diverse repertoire of GPCRs involved in vertebrate olfaction^8^.

Rhodopsin family members involved in vertebrate olfaction include, i) the odorant receptors (ORs)^13^, ii) the trace amine-associated receptors (TAARs)^11^ and iii) the formyl peptide receptor-like proteins (FRPs)^14^. Type 1 and 2 vomeronasal receptors are also GPCRs involved mainly in vertebrate pheromone detection but poorly represented in teleost fishes^15–17^. Teleost fish possess olfactory receptors related to the rhodopsin and glutamate GPCRs involved in sensing of pheromones and other molecules such as amino acids^18–20^. ORs are the largest GPCR subfamily in vertebrates but gene number across species is variable, with some teleost fish genomes possessing fewer than 100 OR genes and some mammals at least 10 times more^2,21^. The uniqueness of the OR subfamily resides in its rapid evolution and the great diversity of receptors which has been fuelled by lineage and species-specific gene duplications and gene loss events^22,23^.

In invertebrates, olfaction appears to be mediated by evolutionary distinct receptors from those of vertebrates^1^. In insects, ORs are not GPCRs as they adopt a distinct membrane topology^24,25^. In other protostomes, GPCR chemoreceptors have been characterised in the nematode *Caenorhabditis elegans*^26^, in the marine mollusc *Aplysia californica*^27^ and in the cnidarian *Nematostella vectensis*^28^, with only the latter showing similar gene structure to vertebrate ORs. In basal deuterostomes, no OR-like genes were found in genomes of the urochordates *Ciona intestinalis*, *C. savignyi* or *Oikopleura doica*^29,30^ and in the hemichordate *Saccoglossus kowalevskii*^31^. However, more than 30 vertebrate-type OR genes have been described in the genome of the cephalochordate *Branchiostoma floridae*^29,30,32^. In echinoderms, a unique OR-like gene repertoire characterized by large groups of independently expanded receptor genes was described in the sea urchin *Strongylocentrotus purpuratus* genome (referred to as the *surreal*-GPCRs)^33^ and in the crown-of-thorn starfish *Acanthaster planci*^34,35^. These receptors have been mostly found in tissues that are in direct contact with the environment such as pedicellariae, spine, tube feet, mouth, body wall and tentacles, although they were also present in internal organs including stomach, testis and radial nerves^33–36^.

Echinoderms, such as the sea cucumbers, are slow-moving and mainly broadcast spawning marine invertebrates without well-developed senses with the exception of mechanoreception, chemosensation and photosensitivity. They largely rely on chemoreception to accomplish their daily tasks such as foraging for food, finding mates, and synchronising their reproductive behaviour^34,37–41^. *Apostichopus japonicus* is the main aquaculture species in China and its genome has been published^42^. *Holothuria arguinensis* is both a recent fishery target from the North-eastern Atlantic Ocean^43^ and a potential species for sea cucumber aquaculture development in Europe^44^. As with most sea cucumbers, it is an important recycler of organic matter^45^. Recently, we have shown that males of this species release chemical cues that attract and induce spawning in ripe male and female conspecifics^46^. However, where and how these cues are detected and what physiological responses they trigger that lead to locomotion towards the cue source, or to initiate spawning, is not known.

The present study therefore aimed to identify the holothurian chemosensory GPCRs repertoire and to characterize receptor tissue distribution in the sea cucumbers *H. arguinensis* and *A. japonicus* as a step towards further understanding of their neurophysiological responses to chemical cues. If sea cucumbers use these receptors to perceive pheromonal signals, it is expected that specific tissues in contact with the environment should be enriched in their transcripts. To test this hypothesis, six transcriptome libraries from tissues of *H. arguinensis* with a potential role in chemosensing (oral cavity, calcareous ring, tentacles and, papillae/tegument) and in reproduction (ovary and testis) were sequenced. Candidate chemosensory GPCRs were retrieved and characterized from the genome and transcriptome of the two species based on a combination of sequence functional annotation, hidden Markov models (HMMs) and phylogenetic analyses. Putative chemosensory receptors were mapped to the different tissue libraries to infer their location.

## Results

### Transcript and assembly annotation

The *H. arguinensis* pooled raw reads from different tissues were assembled into 810,312 contigs with a N50 value of 628 bp. For the individual tissue libraries, the largest and lower number of contigs were found in *de novo* assemblies of the oral cavity (OC; 353,921 transcripts) and ovary (O; 86,417 transcripts), respectively (Table 1).

**Table 1:** Descriptive statistics of the individual and combined tissue assemblies.

	O	Ts	Tt	OC	CR	P + T	Combined
Raw reads (paired end)	51,278,830	61,266,642	62,269,746	43,209,266	38,258,668	44,636,084	300,919,236
Post-QC reads	51,200,570	61,211,616	62,185,582	43,113,778	38,167,808	44,549,154	300,428,508
Total contigs	86,417	153,723	200,418	353,921	257,323	327,647	810,312
N50 (bp)	678	656	726	741	656	691	628
Average length (bp)	587	571	600	590	552	565	518

O: ovary, Ts: testis, Tt: tentacle, OC: oral cavity, CR: calcareous ring, P + T: papillae/tegument, Combined: all tissue combined.

### GPCR transcripts and genes

The *H. arguinensis* transcriptome originated a total of 1,580 contigs with five, six and seven predicted transmembrane domains (TMs), of which 474 were retained as putative GPCRs. After elimination of duplicates, 246 were considered unique GPCRs and 236 were classified into the five main GRAFS families: glutamate (21), rhodopsin (141), adhesion (56), frizzled (3) and secretin (15) (Supplementary Table S1). Searches in the *A. japonicus* genome identified 310 GPCR genes, including 297 that were classified into the five GRAFS families (13 Glutamate, 231 Rhodopsin, 39 Adhesion, 1 Frizzled, 13 Secretin), suggesting that a similar number of receptors exists in the two sea cucumber species (Supplementary Table S2). Members of the vomeronasal and taste 2 receptors were not identified in either species.

The rhodopsin family was the largest and most represented, with more than 50% of the total GPCRs found both in the *H. arguinensis* transcriptome (141 transcripts) and *A. japonicus* genome (231 genes). The receptors within this family belonged to the four main groups represented in human (α, β, γ, δ), and the α group had the most numerous and diversified receptors in both species (60 in *H. arguinensis* and 125 in *A. japonicus*). The MAS-related and the purine receptors, both within the *delta* group, were absent and only a single transcript showed similarity to mammalian OR in *H. arguinensis* (Table 2). In both species, approximately 10% of the rhodopsin GPCRs could not be assigned to a group and were designated as “unclassified rhodopsins” (Table 2, Supplementary Table S1).

**Table 2:** Putative GPCRs found in *H. arguinensis* (Ha) transcriptome and *A. japonicus* (Aj) genome.

Family	Receptor cluster	Number	
		*Transcriptome (Ha)*	*Genome (Aj)*
α - Rhodopsin	Amine	31	78
	MECA	6	8
	Melatonin	6	14
	Opsin	15	22
	Prostaglandin	2	3
β - Rhodopsin	Peptides	38	56
γ - Rhodopsin	Chemokine	3	3
	MCH	1	1
	SOG	13	17
δ - Rhodopsin	Glycoprotein	7	9
	Olfactory	1	0
Other Rhodopsins	Unclassified	18	20
Adhesion	Cadherin	3	1
	Latrophilin	2	0
	Protocadherin	1	0
	Unclassified	50	38
Glutamate	GABA	9	5
	Metabotropic glutamate	10	6
	Unclassified	2	2
Secretin	Methuselah-like	7	4
	CRH	3	1
	CAS	0	2
	CALC	1	1
	PDF	1	2
	PTH	1	2
	Unclassified	2	1
Frizzled	Frizzled	3	1

CALC: calcitonin; CAS: calcium-sensing; CRH: corticotrophin-releasing hormone; GABA: gamma-amino-butyric acid; MCH: melanin-concentrating hormone; MECA: melanocortin, endothelial, cannabinoid and adenosine, PDF: pigment dispersing factor; PTH: parathyroid hormone; SOG: somatostatin, opioid and galanin.

Adhesion was the second largest GPCR families identified in *H. arguinensis* transcriptome (23%) and *A. japonicus* genome (12%). Glutamate (8%) and secretin (6%) were the third and the fourth most abundant family in *H. arguinensis*, while they were equally represented in *A. japonicus* (4%). Metabotropic glutamate and GABA (γ-aminobutyric acid) receptors were identified in the glutamate family in both species but no taste receptors were retrieved. Putative calcium-sensing receptors were only found in the *A. japonicus* genome (Table 2). Methuselah-like were the most abundant receptors in the secretin family in both species, with few corticotrophin-releasing hormone (CRH), parathyroid hormone (PTH), pigment dispersing factor (PDF) and calcitonin (CALC) receptors (Table 2). Members of the frizzled family (<2%), which are involved in tissue polarity and cell signalling in vertebrates were the least represented in both species.

### Identification of OR-like candidates

To select the putative OR-like candidates among the 141 and 231 rhodopsin-like receptors in the *H. arguinensis* transcriptome and *A. japonicus* genome, respectively, HMM analysis was performed against 23 different OR and OR-like protein sequence profiles built from teleost fish, amphioxus, mollusc, sea anemone and echinoderm receptor sequences. Sequence hits were obtained mainly against the echinoderm (starfish and sea urchin) profiles and only a few against the sea anemone profile (Supplementary Tables S3 and S4). No hits were obtained against the profiles of other organisms. In total, 78 transcripts and 112 genes were retrieved as putative OR-like sequences for *H. arguinensis* and *A. japonicus*, respectively.

**Figure 1:**
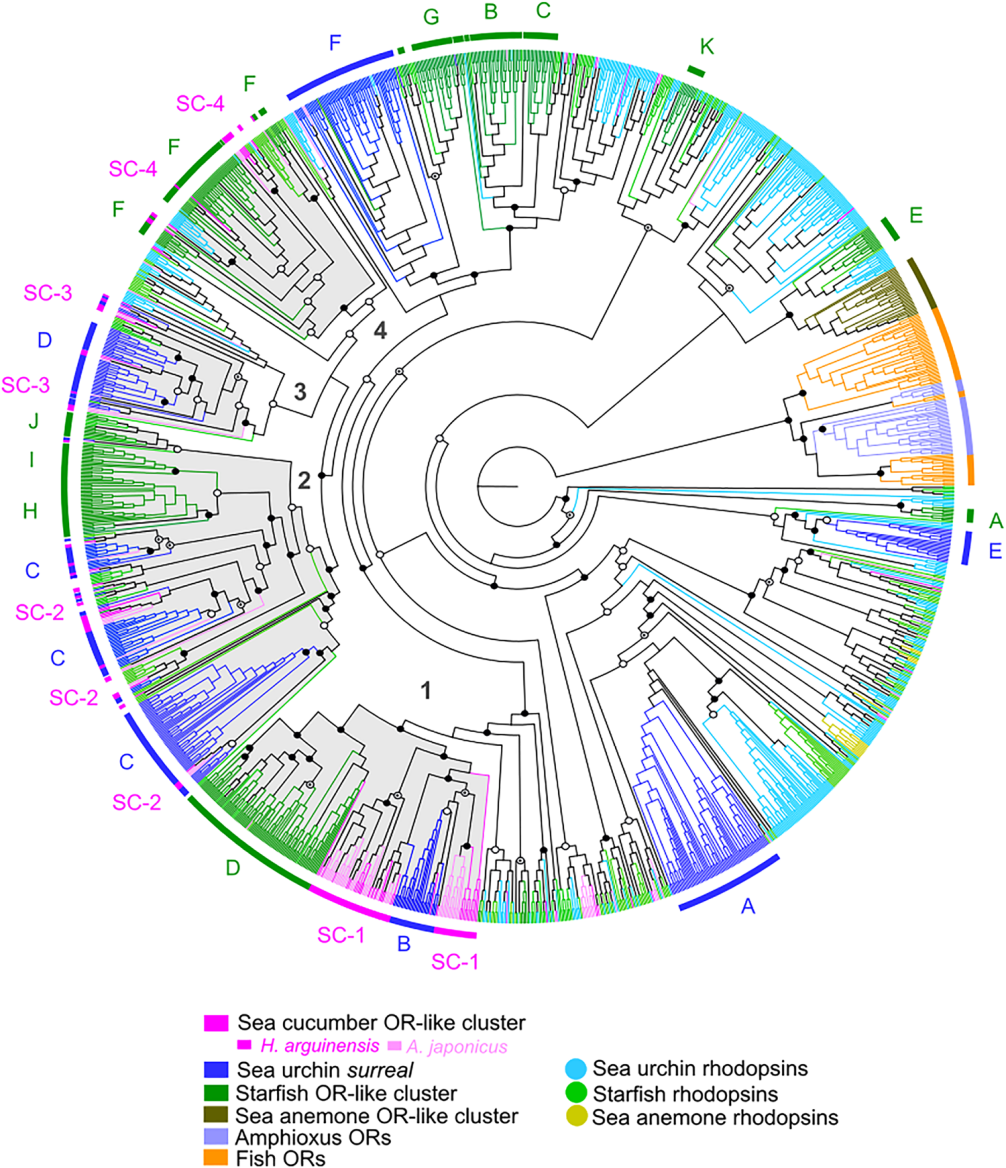
Phylogeny of the sea cucumber OR-like candidates (*H. arguinensis*, 78 sequences and *A. japonicus*, 112 sequences) selected by the HMM analysis with OR-like/olfactory and non-olfactory rhodopsins from other echinoderms (*A. planci*, starfish and *S. purpuratus*, sea urchin), cnidaria (*N. vectensis*, sea anemone) and ORs from cephalochordates (*B. floridae*, amphioxus) and aquatic vertebrates (*O. latipes*, teleost fish). The ML tree was rooted with the chordates (amphioxus and teleost fish) cluster. The different OR-like/olfactory clusters are highlighted by a coloured line, corresponding to the respective group, around the tree. The four echinoderm OR-like clades are shaded in grey and numbered according to the cluster name: 1 is OR-l1, 2 is OR-l2, 3 is OR-l3 and 4 is OR-l4. The sea cucumber specific gene expansions are designated as SC-1, SC-2, SC-3 and SC-4. Branch support was represented only when at least one of the three methods used (aLRT-Chi^2^, aBayes and SH-LRT) had statistically significant supporting values. Tree branch symbol: full circle: three methods were significant; circle with a dot: two methods were significant and empty circle: one method was significant. SC: sea cucumber.

Phylogenetic analysis revealed that most of the sea cucumber putative OR-like sequences cluster in proximity with the sea urchin and starfish OR-like sequences and originate four main receptor sequence clades (Fig. 1). Each of the four OR-like echinoderm clades was statistically supported by all the three branch support methods used (i.e. aLRT-Chi^2^, aBayes and SH-LRT) and were designated as OR-l1, OR-l2, OR-l3, OR-l4. The tree topology also suggested that the echinoderm OR-like groups shared common ancestry and that the clade OR-l1 was the first to diverge followed by OR-l2, OR-l3 and OR-l4 in subsequent duplication events. Gene expansions that resulted from species-specific events were also observed specially within the OR-l1 cluster (denoted as SC-1 in Fig. 1).

**Figure 2:**
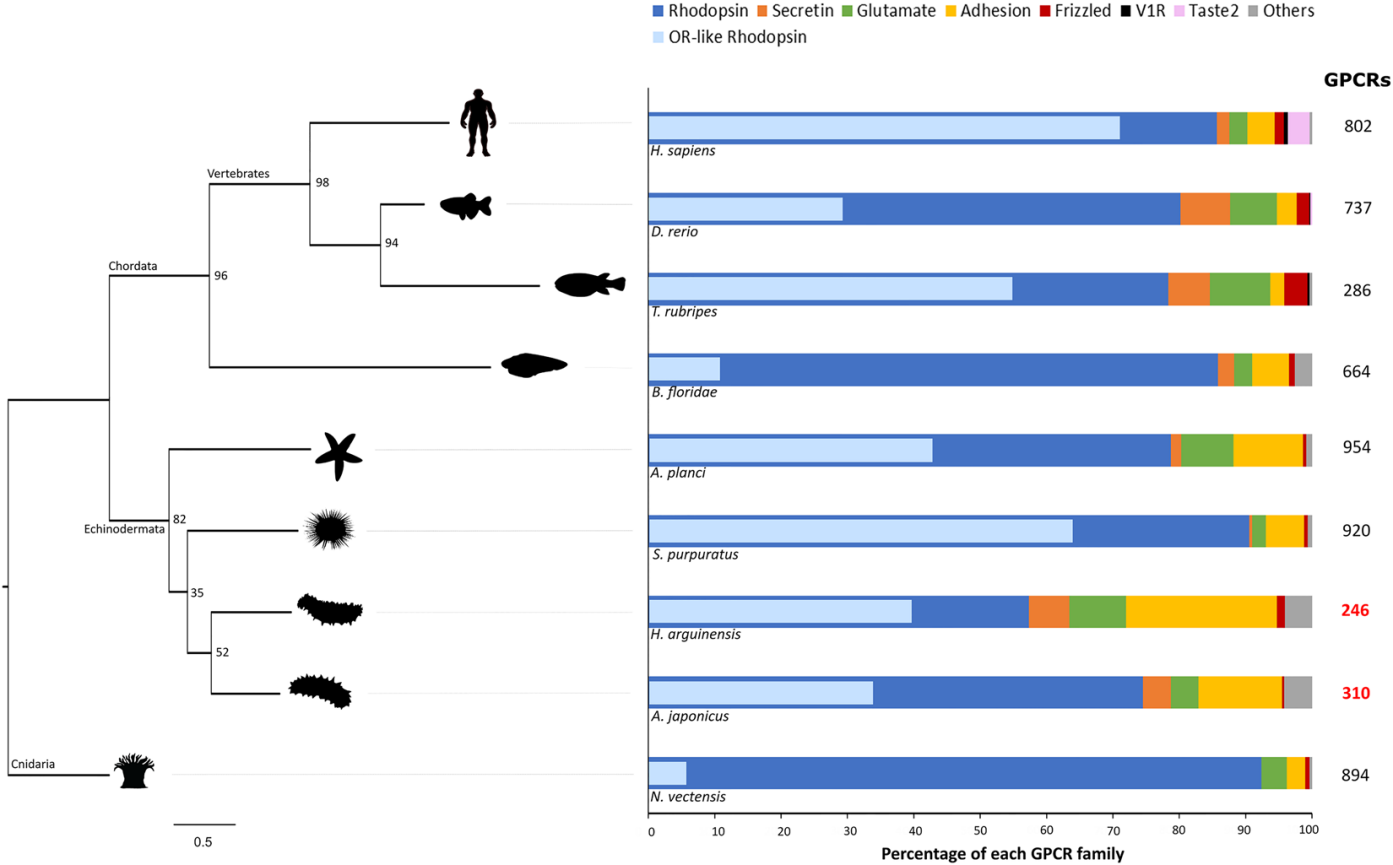
Number and percentage of GPCRs, including the percentage of OR-like within the Rhodopsin family, in different species: *N. vectensis*^28,47^*, A. japonicus* and *H. arguinensis* (present manuscript)*, S. purpuratus*^33,47^ (ORs: *surreal*-GPCRs; groups A-F)*, A. planci*^34^ (ORs: groups A-K)*, H. sapiens*^47,48^, *D. rerio*^30,48^*, T. rubripes*^30,48^ and *B. floridae*^29,86^. The cladogram corresponds to a species tree that was built using the ML method with concatenated sequence of four ORs per species. The values of the bootstrap are seen at the nodes of the trees. This species tree is in agreement with the generic tree defined in the Tree of Life^93^ (http://tolweb.org). The percentage of OR-like Rhodopsin found within the Rhodopsin family is represented in light blue.

In total, 57 *H. arguinensis* and 79 *A. japonicus* GPCRs, which clustered with other echinoderm OR-like sequences, were retained as OR-like candidates (Fig. 1; Supplementary Tables S1 and S2). Clade OR-l1 grouped 32 *H. arguinensis* and 44 *A. japonicus* OR-like candidates (SC-1 in Fig. 1) with sequences from the sea urchin *surreal*-GPCRs group B and starfish OR-like group D. Clade OR-l2 contained 11 and 19 OR-like sequences from *H. arguinensis* and *A. japonicus*, respectively (SC-2 in Fig. 1), which clustered with the sea urchin *surreal*-GPCRs group C and the starfish OR-like groups H, I, J. The clades 3 and 4 contained the least number OR-like sequences: 6 from *H. arguinensis* and 8 from *A. japonicus* grouped with the sea urchin *surreal*-GPCRs group D (SC-3 in Fig. 1); and 8 from *H. arguinensis* and *A. japonicus* clustered with the starfish OR-like group F (SC-4 in Fig. 1). Figure 2 summarizes the number of GPCRs and their family members, including olfactory receptor in vertebrates and invertebrates.

The amino acid sequence similarity and identity between the *H. arguinensis* sequences within the same OR-like clade was on average 41% and 24%, respectively (Supplementary Table S5). When these sequences were compared with the homologues from other echinoderms, clade OR-l1 was the most conserved with 43% and 25% of similarity and identity, respectively, while the clade OR-l3 was the least conserved (33% of similarity and 20% of identity). Between the four clades the average similarity and identity of *H. arguinensis* OR-like was much lower, respectively, 26% and 12%, with the lower values between OR-l1 and OR-l3 (20% similarity and 9% identity) and the highest values between OR-l1 and OR-l2 (31% similarity and 15% identity).

As a measure of the reliability of our methodology, we estimated the number of GPCRs/ORs in the sea urchin *S. purpuratus* and in the teleost *Takifugu rubripes* using the same bioinformatics pipeline and compared with the numbers found in the literature for these species. In *S. purpuratus*, our method identified 954 GPCRs of which 695 were putative ORs. These numbers are similar to the published estimates − 920^47^ to 979^33^ GPCRs of which 538 (*surreal*-GPCRs)^33^ were chemoreceptors. Similarly, the number of GPCRs and ORs found in *T. rubripes* with our method were 328 and 248 respectively, not very different from those found in previous studies − 286^48^ to 298^49^ GPCRs of which 125 were identified as ORs^30^.

### Genome mapping of OR-like genes

**Figure 3:**
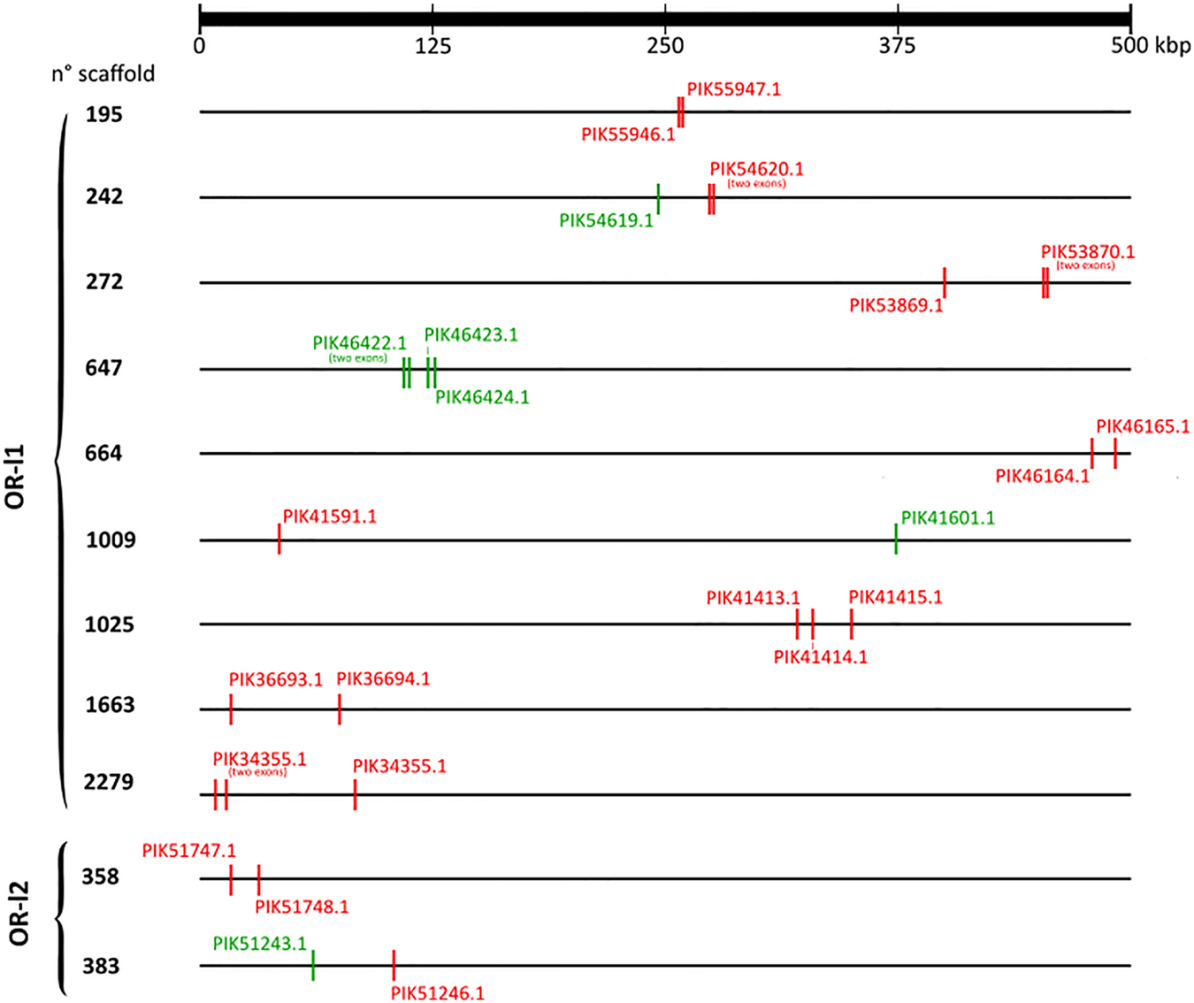
Mapping of the OR-like gene candidates in *A. japonicus* genome. Only scaffolds containing at least two OR-like genes are represented. Horizontal lines represent the genome fragments and each vertical coloured line represents an exon. There are putative sea cucumber OR-like genes with two exons. Gene orientation is denoted by colour: genes in the sense strand are represented in green and genes in the antisense strand are represented in red. The bar on top indicates the absolute distance in kilo base pairs (kbp).

Analysis of the *A. japonicus* genome revealed that several OR-like receptor genes belonging to OR-l1 and OR-l2 were organized in tandem arrays (Fig. 3). However, no OR-like candidates from OR-l3 and OR-l4 were seen on the same genome scaffold.

### Effect of selection on putative OR-like receptors

To get an insight into the mechanisms that shape evolution of sea cucumber OR-like receptors, likelihood ratio analysis was carried out based on the ratios of non-synonymous (modifying amino acids) versus synonymous (non-modifying or silent) substitutions of the four OR-l clades. The likelihood ration test (LRT) for the branch (M0:M1) specific analysis revealed that for three (1, 3 and 4) out of the four OR groups, the data had a better fit to the free-ratio model (M1) where ω may vary between the branches indicating variable selective pressure regimes in each branch. The results for the site models (M7:M8) indicated that the four OR groups best fitted the M7 model which only allows for ω ≤ 1. Accordingly, the estimated ω values under the M8 model (Table 3) were significantly less than one, hereby indicating purifying (negative) selection among the different sites for each receptor clade (Table 3; Supplementary Note).

**Table 3:** Likelihood Ratio Tests (LRT) of selective pressure on OR-like receptor clades.

Group	n	P-value M0:M1	P-value M7:M8	M8 ω Estimates
1	44	**<0.00E + 00**	9.80E-01	0.1247
2	14	2.03E-02	9.97E-01	0.2182
3	10	**<4.30E-05**	6.77E-01	0.2489
4	14	**<9.00E-09**	9.95E-01	0.2292

The P-values were calculated from the χ2 distributions; P-values in bold represent statistically significant tests in which the free-ratio model (M1) was a better fit than the one-ratio model (M0) and are Bonferroni corrected for the number of sequences in each group with α = 0.05; n= Number of sequences in the group; ω is *dN:dS* estimated under the M8 model.

### Echinoderm OR-like signature motifs

Several motifs have been found that discriminate ORs from non-ORs: LxxPxYxxxxxLxxxDxxxxxxxxP, MAxDRYxxxCxPLxY, KAxxTxxxH and PxxNPxxY (where x is any amino acid) which are conserved amongst vertebrates^28,29,50^. Analysis of the entire sea cucumber OR-like repertoire revealed that several amino acids were conserved in the LxxPxYxxxxxLxxxDxxxxxxxxP (intracellular loop, IL, 1)/ transmembrane domain, TM, 2), MAxDRYxxxCxPLxY (TM3/IL2) and PxxNPxxY (TM7) motifs (Supplementary Fig. S1). However, the KAxxTxxxH motif (IL3/TM6) was poorly represented in sea cucumber ORs, where only the lysine (K) was conserved, as seen also in cnidaria.

**Figure 4:**
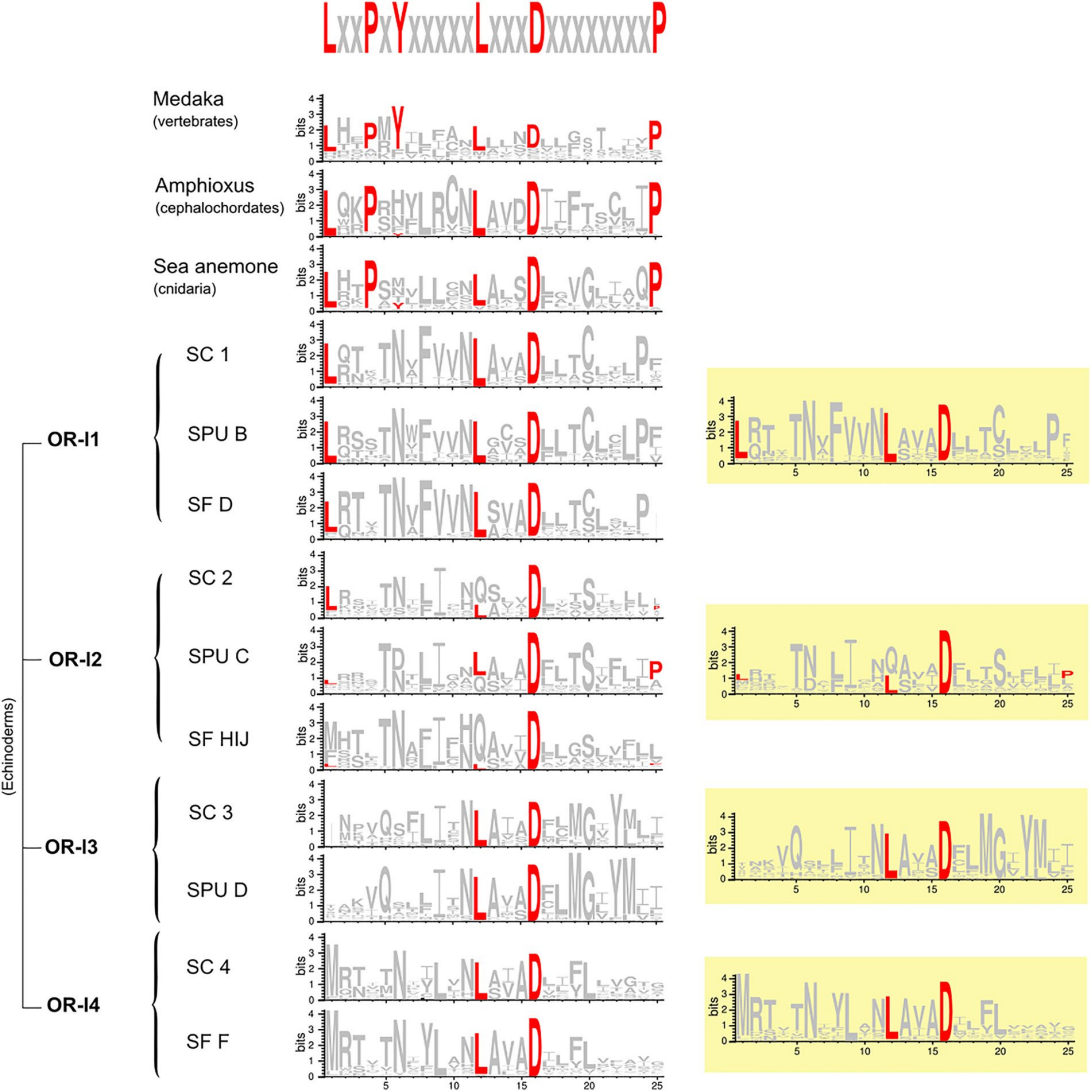
Weblogo obtained from the alignment of the motif LxxPxYxxxxxLxxxDxxxxxxxxP. A consensus echinoderm weblogo resulting from the alignment of all sequences identified per echinoderm cluster is represented with the yellow background. The height of each symbol indicates the relative frequency of each amino acid at that position.

Four amino acid residues (alanine, A; arginine, R, tyrosine, Y; proline, P) within the MAxDRYxxxCxPLxY motif and three (asparagine, N; second proline, P; tyrosine, Y) within the PxxNPxxY motif were commonly found in sea cucumber ORs. Because these two motifs might not be specific to ORs due to the presence of the DRY and NPxxY residues that are characteristic to the rhodopsin-like family^34^, the LxxPxYxxxxxLxxxDxxxxxxxxP motif was choosen as OR target in sea cucumbers, as previously described for other echinoderms (starfish^34^ and sea urchin^28^). This motif was searched in each of the phylogenetic tree clades and a consensus motif sequence was determined for each of the four echinoderm OR-like clades. In amphioxus and sea anemone, the OR motif contained five of the six conserved residues, with the tyrosine (Y) typical of vertebrates OR motifs largely absent (Fig. 4). In the echinoderm OR-l1 clade, three residues including the two lysines (L) and the aspartic acid (D) were present (LxxxxxxxxxxLxxxD) while aspartic acid (D) was the dominant residue in OR-l2. In OR-l3 and OR-l4, the second lysine (L) and aspartic acid (D) were preserved over the six conserved residues (seen as LxxxD).

### Tissue expression of the OR-like candidates

**Figure 5:**
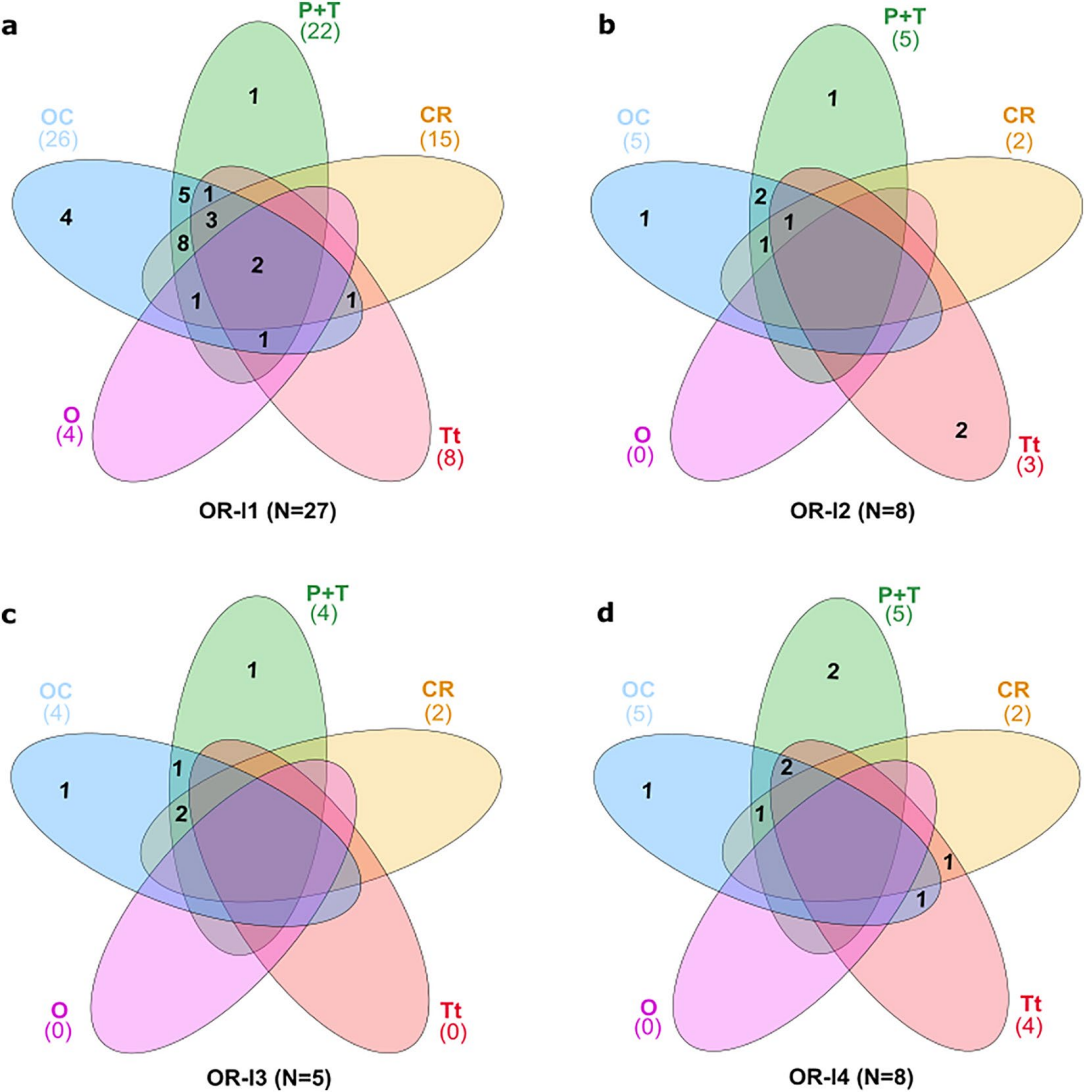
Venn diagrams showing the distribution of the OR-like candidates from each cluster (**a**: OR-l1, **b**: OR-l2, **c**: OR-l3, **d**: OR-l4) among tissues. OC: oral cavity, P + T: papillae/tegument, CR: calcareous ring, Tt: tentacles, O: ovary). N = number of OR-like receptors found in each clade.

Forty-eight of the 57 OR-like candidates selected through the combination of the HMM profiles and phylogenetic analysis were mapped in the six tissue transcriptomes of *H. arguinensis* (Supplementary Table S6). At least 75% of the 48 OR-like candidates identified were found to be present in the oral cavity (36) and the papillae/tegument (40) followed by the calcareous ring (21) and the tentacles (15), which represented 35% of the OR-like candidates mapped. None of the OR-like candidates were found in the testis and only four were mapped in the ovary (less than 10% of the OR-like candidates). The calcareous ring, the papillae/tegument, and the oral cavity expressed members of all the four echinoderm OR-like candidate clades. However, OR-l3 members were not found in tentacles, and the ovary contained only a subset of OR-l1 members (Fig. 5). Most receptors within each cluster overlapped among tissues, with a few unique receptor transcripts identified in oral cavity and papillae/tegument. More OR-like transcripts were found in the oral cavity (113.02 transcripts per million, TPM) and papillae/tegument (109.88 TPM) followed by the calcareous ring (81.61 TPM), tentacles (74.84 TPM) and ovary (46.29 TPM).

**Figure 6:**
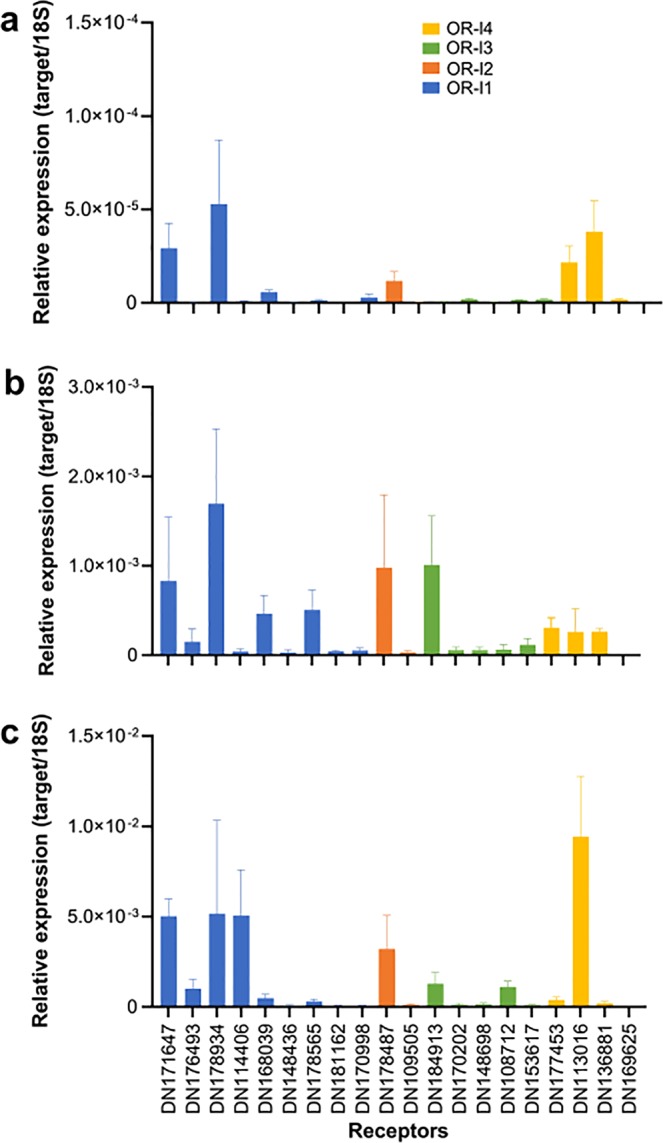
Expression of 20 *H. arguinensis* OR-like gene candidates from the four echinoderm OR-like groups analysed by qPCR in (**a**) tentacles, (**b**) papillae/tegument, (**c**) oral cavity. Data are presented as the mean ± SEM (n = 3 biological replicates). Notice the difference in scale in the ordinates.

Quantitative reverse-transcription polymerase chain reaction (qPCR) of 20 selected OR-like candidates confirmed generally higher levels of expression in the oral cavity and papillae/tegument and lower levels in the tentacles (Fig. 6). These receptors belonged to the four OR-like groups, and some (i.e. DN171647, DN18934, DN14406, DN113016) were 2–3 orders of magnitude more highly expressed in the oral cavity than in the tentacles (Fig. 6).

## Discussion

A large diversity of GPCRs, of which 57 were considered OR-like, were identified in the *H. arguinensis* transcriptome and a slightly higher number (79 OR-like genes) were retrieved from the *A. japonicus* genome. These OR-like candidates were organized in four main clades that cluster with other echinoderm OR-like sequences from starfish and sea urchin, with each clade showing a characteristic signature motif. Most of these OR-like candidates were found in sensory tissues including tentacles, oral cavity, calcareous ring, and papillae/tegument, which is consistent with their potential involvement in chemical sensing.

The number of *H. arguinensis* and *A. japonicus* GPCRs (respectively 246 and 310 receptors) is of the same order of magnitude as reported for the hemichordate *S. kowalevskii* (260)^31^, the urochordate *C. intestinalis* (169)^51^ and the demosponge *Amphimedon queenslandica* (220)^52^. However, this number is smaller than in other echinoderms which genomes possess more than 900 GPCRs such as the starfish *A. planci*^34^ and sea urchin *S. purpuratus*^33^. This suggests that sea cucumbers have a more compact GPCR gene repertoire than starfish or sea urchin.

Odours are mostly detected by rhodopsin-like GPCRs^2^. However, no orthologues of the TAARs, FRPs and vomeronasal 1 and 2 receptors, characteristic of vertebrate olfaction, were identified in the sea cucumber. Neither were they found in other invertebrates such as starfish^35^, sea urchin^33^, sponge^52^ or mollusc^27^. In contrast, based on HMM profiles and phylogeny, 57 *H. arguinensis* transcripts were considered OR-like and grouped in four OR-like clades with the starfish and sea urchin OR-like genes. Within these clades, there were large independent OR-like gene expansions that resulted from species-specific events. They were found mainly within the OR-l1 cluster, which is consistent with what has been described for the starfish and sea urchin ORs^33–35^. These lineage specific OR-like gene expansions could be linked to the rapid evolution and diversification of this receptor group as described in chordates^53–56^. They are also characterized by a relatively low percentage of sequence similarity and identity between the sea cucumber and other echinoderm OR-like sequences within each clade (*ca*. 22% of identity and 39% of similarity). The fact that sea cucumber OR-like receptor genes are mostly single exon and are organized in tandem arrays, as seen also in vertebrate ORs^6,13^ and other invertebrates^27,33,35^, supports their olfactory receptor nature. Interestingly, the olfactory receptor signature sequence motif “LxxPxYxxxxxLxxxDxxxxxxxxP” was differently conserved across the four OR-like clusters and was most conserved in ORl1. Whether changes in receptor sequence are linked with their functional divergence deserves further investigation.

Phylogenetic analysis revealed that the sea cucumber OR-like receptors emerged early in evolution and shared common ancestry with the other echinoderms. Evolution of the sea cucumber OR-like genes is likely to be the consequence of gene duplications that occurred earlier in the radiation of the echinoderms and to more recent duplications that independently occurred in Holothurians. Analysis of selection in OR-like receptors through the ratio of nonsynonymous to synonymous changes suggest that they are under purifying selection (ω < 1), which presumably maintains their function intact and reduces genetic variation. This is in agreement with several studies in vertebrates and *Drosophila*^57^, which are in general under weak purifying selection with no evidence for positive selection^58–63^. The extensive variation of OR genes among species, increasing rapidly in some species and undergoing mass pseudogenization in others suggests that olfactory ability is probably linked to the diversity of OR gene repertoires and their level of expression^53^.

In marine invertebrates, the OR repertoire seems to be variable between different taxonomic groups. No ORs have been found in urochordates, hemichordates and porifera^31,51,52^; however, at least 300 OR-like were seen in starfish and sea urchin^33,34^. Compared to the rest of the echinoderms, sea cucumbers seem to have the smallest OR-like repertoire as shown in our transcriptome (57 transcripts) and genome (79 genes) analysis. The total number of OR-like transcripts in *H. arguinensis* could, nevertheless, have been underestimated due to our restrictive approach to select only GPCRs with at least five TMs and some GPCRs are known to be expressed only in larval stages^33,36^. Other sources of errors might have come from the HMM profiles generated, the transmembrane domain prediction or the phylogenetic clustering. Some putative ORs might also have been missed in our transcriptome due to assembly errors (formation of chimeras and fragmented contigs) even though this was limited by using the whole tissues transcriptome. However, only 22 additional OR-like genes were found in *A. japonicus*, suggesting that sea cucumbers possess fewer olfactory receptors than sea urchins and starfish. Moreover, the number of GPCRs and ORs obtained in *S. purpuratus* and *T. rubripes* with our methodology was similar, although slightly higher (and not smaller), to those found in previous studies. This increase is likely to be due to the integration in our analysis of more up-to-date models that incorporated recent data from other invertebrate OR-like sequences such as those from the cnidarian *N. vectensis*^28^ and the phylogenetically related echinoderm *A. planci*^34^. Also, we have considered GPCRs containing 5 to 7 TMs while other studies, such as Raible, *et al*. (2006)^33^, included only 7 TMs sequences. It is also important to note that chemosensory receptors other than GPCRs may also exist in sea cucumbers. For example, putative variant ionotropic glutamate receptors were recently discovered in the starfish *A. planci*^64^.

The specific function of ORs in echinoderms is unknown as the vast majority of putative ORs are orphans. Also, it is not known if there are differences on how the different classes of echinoderms perceive the environment. Anatomically, sea urchins and starfish are the only ones to possess pedicellariae in addition to the tube feet that is common to all echinoderms. These appendages are mainly involved in defense mechanisms and are divided in four different types which are thought to harbour different chemoreceptors as they react to different chemical stimuli^65^. The OR-like transcripts identified were largely found in three tissues that are in direct contact with the environment - the tentacles, oral cavity and papillae/tegument – which have been previously described as sensory tissues^66,67^ and qPCR of 20 OR-like confirmed that they are orders of magnitude more highly expressed in the oral cavity and papillae/tegument, supporting their potential role as chemosensory organs in sea cucumbers. This draws a parallel with the putative chemoreceptors found in body appendages, mouth and tegument in starfish^34,35^ and sea urchin^33,36^. However, OR-like candidates were also found in sea cucumber internal tissues (46% of the total) such as the calcareous ring that is connected to the radial nerve cord, and in ovary. The presence of OR-like candidates has also been reported in testis, sperm, oocytes, radial nerves, stomach in other echinoderms^33–35,68^. These findings are not surprising as in vertebrates ORs have been found in non-chemosensory organs such as testes and sperm, lung, spleen, liver, heart and thyroid where they are thought to carry out diverse and unrelated functions, not specifically linked to sensorial perception, which include cell-cell communication, chemotaxis and tissue regeneration^69–71^.

## Conclusion

As sea cucumbers have limited visual abilities and no hearing; they rely more on chemosensation for detecting biotic and abiotic factors in their environment. The sea cucumber GPCR repertoire, of which 20% are OR-like, is extensive but less expanded than in other echinoderms. The OR-like receptors from sea cucumber grouped with other echinoderm receptors into four distinct clades, which suggests that each clade may be involved in different functions. These receptors are under purifying selection and receptors from the OR-l1 clade are the most conserved and potentially the most interesting candidates as pheromone receptors. They have a widespread distribution and are mostly expressed in tissues that are in direct contact with the external environment such as tentacles, oral cavity and papillae/tegument. Our results provide the first molecular basis of chemical sensing in sea cucumbers which is an essential step to the understanding on how these animals communicate.

## Materials and Methods

### Ethics statement

The specimens of the sea cucumber, *Holothuria arguinensis*, Koehler & Vaney, 1906 (Holothuroidea, Aspidochirotida), were collected and handled in agreement with the license of the ICNF, Instituto da Conservação da Natureza e das Florestas, Portugal (License N° 635/2015/CAPT). This species is not endangered or protected.

### Collection of animals and tissues

Adult, reproductively mature, *H. arguinensis* (> 210 mm length) were hand collected in the intertidal zone of the Ria Formosa (37°00′35.02″N; 7°59′46.10″O) in Faro (Portugal) during summer 2015 and transported live in individual plastic bags filled with natural sea water to the Centre of Marine Science (CCMAR), University of Algarve. After arrival at the laboratory, they were anesthetized by immersion in MgCl_2_ (5%) in sea water and tissue samples (tentacles, testis and ovary, papillae and tegument, oral cavity and calcareous ring, including nerve ring) were dissected and pooled from four individuals (two males and two females, except for gonads in which four individuals of each sex were pooled). All samples were frozen on dry ice and kept at −80 °C until RNA extraction.

### RNA extraction and library preparation

Total RNA was extracted using the Maxwell 16 total RNA purification kit (Promega, Madrid, Spain) according to the manufacturer’s instructions. Pooled samples were homogenized using an Ultra-Turrax homogenizer (IKA T25, Staufen, Germany) and total RNA was precipitated with ethanol and quantified using a Nanodrop (1000 Spectrophotometer, Thermo Fisher Scientific, USA). Agarose gel electrophoresis (0.8%/1 × TAE: Tris-acetate-EDTA) was used to assess the RNA quality and integrity. RNA samples were subsequently treated twice with DNAse to remove any remaining genomic DNA using the Turbo DNA-free kit (Ambion, London, UK).

Sequencing library preparation and sequencing was conducted by Genenergy (Shanghai, China) using a Illumina TrueSeq mRNA-seq library Prep kit (RNA input 2 µg, insert size of 300–400 bps) and sequenced using the Illumina Hi-Seq. 1500 to generate 100 base paired-end reads (Bioproject Accession no.: PRJNA532556).

### Sequence assembly

Quality control of raw reads and their respective editing was performed with Trimgalore wrapper script version 0.3.3^72^ producing simple descriptive statistics and edited reads, before assembly. Tissue specific *de novo* assemblies were obtained using Trinity v. 2.0.6 (trinityrnaseq_r2012-05-18) with the default parameters^73^. The pair-end reads from each of the six tissue libraries were used to assemble tissue specific *de novo* transcriptomes. In order to increase the probability of retrieving low expressed transcripts, a whole tissues transcriptome was also created using the pooled pair-end reads of all the tissues and Trinity with “–normalize_reads” and “–min_kmer_cov 2” options defined.

### GPCR sequence annotation

The whole tissues *de novo* transcriptome from *H. arguinensis* was translated into protein using the TransDecoder v5.0.2^74^. The predicted proteins were annotated using TMHMM v2.0^75^ to generate a sub-library containing transmembrane (TM) domains proteins. Only sequences with predicted five to seven TM domains were considered and were further annotated using BLASTP v2.7.1+ searches^76^ against Swiss-Prot (version 2018 with 558,125 entries) and Pfam (version 32.0 with 17,929 entries) with a cut-off e-value ≤ 1e^−5^. The *H. arguinensis* GPCRs were then classified into the five main GPCR families and categorized into subfamilies as described by Fredriksson, *et al*.^8^. As a comparison, the GPCR gene repertoire in the *A. japonicus* genome^42^ were annotated the same way as for *H. arguinensis* using as database the predicted proteins of this species (30,221 sequences) available in NCBI (MRZV00000000.1; PRJNA354676).

### Identification of the OR-like candidates: hidden markov models

To identify putative OR-like protein sequences, 23 OR and OR-like profile Hidden Markov Models (HMMs) were built using the hmmbuild of HMMER v.3.1.b2^77^ according to the methodology used to identify OR-like in the starfish *A. planci* and described in Hall, *et al*. (2017)^34^. The HMM profiles were based upon the alignments of teleost fish ORs (*Danio rerio*, *Oryzias latipes*, *Takifugu rubripes*, *Tetraodon nigroviridis*, *Gasterosteus aculeatus*)^30^, amphioxus ORs (*Branchiostoma floridae*)^29^, marine gastropod mollusc chemoreceptors *(Aplysia californica;* groups a to c)^27^, cnidaria OR-like *(Nematostella vectensis*)^28^, sea urchin chemoreceptors (*Strongylocentrotus purpuratus*; *surreal*-GPCRs; groups A to F)^33^ and starfish OR-like (*A. planci*; groups A to K)^34^. Only *H. arguinensis* and *A. japonicus* proteins that aligned to the HMMs using hmmsearch with an *e-value* ≤ 1e^−5^ were selected as OR-like candidates. To estimate the reliability of the methodology used here to identify GPCRs and putative ORs, the same procedure was applied to the sea urchin *S. purpuratus* and the teleost *T. rubripes*, and compared with the published GPCRs/ORs repertoire estimates for these species.

### Identification of the OR-like candidates: Phylogenetic analysis

To select the most likely sea cucumber (*H. arguinensis* and *A. japonicus*) OR-like candidates, a phylogenetic analysis was conducted with the sequences retrieved from the HMM profiles and the olfactory/OR-like rhodopsins and non-olfactory rhodopsins from the cnidarian sea anemone *N. vectensis*^28^ and basal deuterostomes starfish *A. planci*^34^ and the sea urchin *S. purpuratus*^33^; the cephalochordate amphioxus *B. floridae*^29^ and the vertebrate teleost fish *O. latipes*^30^. The final dataset used to build the tree consisted of an alignment performed using Aliview^78^, of 1626 sequences that was subsequently manually annotated to remove gaps and trimmed to conserved transmembrane domains. Due to the large number of sequences, TM domains were initially predicted, extracted and concatenated for ten sequences of each species and each group (i.e. olfactory/OR-like rhodopsins and non-olfactory rhodopsins) using SMART^79^ which were then used as a reference to delete the non-TM regions from the remaining of the sequences in the alignment.

With the edited sequence alignment, a maximum-likelihood (ML) tree was built using PhyML v3.0^80^. The ML tree was constructed using a VT substitution model with a gamma shape (4 rate categories) of G = 1.808, as selected by the SMS (Smart Model Selection)^81^ according to the Akaike Information Criterion (AIC)^82^. The tree was edited in Figtree^83^ and the chordate cluster (amphioxus and fish) was used to root the tree. Branch support was estimated using three methods: two parametric methods, aLRT Chi^2^-based (approximate likelihood ratio test) and aBayes (approximate transformation Bayes test), and one non-parametric method SH-aLRT. The nodes were reported when at least one of the three methods showed significant branch supported values, defined as aBayes ≥ 0.95, aLRT Chi^2^-based ≥ 0.9 and SH-aLRT ≥ 0.85^84^. Sequence identity and similarity (only with the full length receptors) were determined using GeneDoc software^85^.

The percentage of each GPCR family and the percentage of OR-like Rhodopsin within the Rhodopsin family were compared between the sea cucumbers (*H. arguinensis* and *A. japonicus*; present study) and other vertebrates and invertebrates: *Homo sapiens*^47,48^, *D. rerio*^30,48^, *T. rubripes*^30,48^, *B. floridae*^29,86^, *S. purpuratus*^33,47^ (ORs: surreal-GPCRs; groups A-F)*, A. planci*^34^ (ORs: groups A-K), and *N. vectensis*^28,47^. A species tree was built using as input the edited alignment obtained by concatenating the predicted protein sequence of four OR sequences per species using the ML method in the PhyML v3.0 program with 100 bootstrap replicates. The echinoderm OR sequences came from OR-l1 (2 sequences) and OR-l2 (2 sequences) as all species are represented in these clusters.

### Genome mapping of OR-like candidates

To determine if sea cucumber OR-like candidates were positioned in tandem in the genome, the *A. japonicus* OR-like candidates were searched (tBLASTn) against its own genome assembly and the position of OR genes were mapped.

### Selection of putative OR-like receptors

The levels of functional constraint and functional divergence of OR-like receptors were analysed through the ratio of non-synonymous (*dN*) to synonymous (*dS*) substitutions (*ω* = *dN/dS*). Neutral evolution is defined when *ω* = 1, while *ω* > 1 and *ω* < 1 indicate positive (diversifying) and negative (purifying) selection, respectively^87^. The full-length nucleotide coding sequences of the four OR-l clades from the two sea cucumbers were aligned to obtain multiple codon alignments using PAL2NAL v14^88^. These alignments were used to build a phylogenetic tree for each OR-l clade using the ML method in MEGA 7^89^. The codon alignments and their respective phylogenetic trees were then used to calculate the codon substitution ratio (*ω* = *dN/dS)* with the CODEML package in PAML version 4.8^87^. The following branch (M0:M1) and site models (M7:M8) were used^87^ following the methodology of Mondragón-Palomino, *et al*.^90^: the one ratio model (M0) and the free ratio model (M1), the beta model (M7) and the beta- *ω* model (M8) (see Supplementary Note for more information).

### Echinoderm OR-like signature motifs

A WebLogo^91^ using the entire sea cucumber OR repertoire was built to identify putative OR motifs and to highlight conserved amino acid residues that are commonly found in fish, cephalochordates and cnidaria ORs (Supplementary Fig. S1). Previous searches in echinoderms identified conserved amino acids from the OR motif LxxPxYxxxxxLxxxDxxxxxxxxP in sea urchins and starfish^28,34^, and this motif was used as target to identify OR-like motifs in our sea cucumber sequences. Another WebLogo^91^ was created using the multisequence alignment of each receptor clade previously defined in the phylogenetic analysis and the conserved amino acids specific to the target motif were highlighted.

### Tissue expression of OR-like candidates

The OR-like candidates identified in *H. arguinensis* were sought in the six individual tissue assemblies using tBLASTn. Sequences that produced hits with an *e-value* cut-off of < 1e-80, a sequence identity ≥ 97% and a sequence coverage ≥ 150 nucleotides between the query and the subject were considered to be similar. Venn diagrams were created using the web-based tool InteractiVenn^92^ to analyse the distribution of the OR-like candidates from each cluster among the tissue libraries. The proportion of OR-like was estimated by dividing the number of each putative OR contigs by the total number of contigs in each tissue.

qPCR was used to confirm the expression of 20 genes from the four OR-like groups identified in tentacles, oral cavity and papillae/tegument of *H. arguinensis*. Total RNA (tRNA) was extracted from tissues from three adult *H. arguinensis* using the E.Z.N.A kit (VWR, USA) according to the manufacture instructions. DNase I treatment was performed directly on the columns. Primer pairs specific for each transcript were designed using the NCBI primer BLAST and amplicon sizes were between 120 and 200 base pairs.

cDNA was synthesised in 20 µl reactions containing 300 ng of DNase-treated tRNA, 200 ng of random hexamers (Jena Biosciences, Germany), 2 mM dNTPs, 100 U of RevertAid reverse transcriptase and 8 U of RiboLock RNase Inhibitor (Fermentas, Thermo Fisher) for 10 min at 25 °C, 60 min at 42 °C, and 10 min at 70 °C. qPCR reactions were performed on a CFX Connect^TM^ Real-TIME PCR Detection System (Bio-Rad) using 96-well micro plates (Axygen). Reactions were performed in duplicate ( < 5% variation between replicates) in a final volume of 10 µl containing 2 µl of 1:5 and 1:5000 diluted cDNA for target genes and 18 S, respectively, SsoFast EvaGreen Supermix (Bio-Rad, Portugal) and 300 nM of the forward and reverse specific primer. Optimized conditions consisted of: 95 °C for 30 s, followed by 45 cycles of 95 °C for 5 s and 10 s at the appropriate annealing temperature for primers (Table 4).

**Table 4:** Primer sequences, amplicon sizes and annealing temperatures (Ta) used in the qPCR analysis.

Transcript name	OR-like group	Fw/Rw	Primer sequence (5'−3')	Amplicon (bp)	Ta (°C)
DN178565|c0_g4_i1	1	Fw	CTCCAATCGGGCAGCAAGTA	170	58
		Rv	AGCGGAATGAAGGTGCGTTA		
DN181162|c0_g2_i1	1	Fw	GATCACAAGACACGGCACCT	122	56
		Rv	GCGTTGGAGGCATTGGTTTC		
DN170998|c0_g1_i1	1	Fw	TCCACGACTTTGCCTGTGTT	164	58
		Rv	GCAGGAGCCCACCTACCTAT		
DN168039|c0_g1_i2	1	Fw	CCCGAAGTGTTCTGCTCCTC	185	56
		Rv	CCCGTGCAATCCTACACACT		
DN148436|c0_g1_i1	1	Fw	TCGGCTGTGGCAAGATTGAT	173	58
		Rv	GCAGGAGGGGTTTACGTTGT		
DN171647|c0_g1_i2	1	Fw	GTAGTTTCCCCATCCGCCAA	190	58
		Rw	GCAAGACGAACAGGTAGGGT		
DN176493|c0_g2_i1	1	Fw	CAGACGACGACAAGCCGATA	199	58
		Rw	AGGGCACACCAAGGAATAGC		
DN178934|c0_g6_i1	1	Fw	GGGAAGAGGTGATCCGAACG	170	62
		Rw	CGTCGTTACTGCTATGGGGG		
DN114406|c0_g1_i1	1	Fw	AGATTCTTGACGCTGCTCCG	151	58
		Rw	CTTGAGTTGAGGGGTCGCAT		
DN109505|c1_g1_i1	2	Fw	ATCGCCCGTTCACACATACC	199	56
		Rv	TCGCTTCTAGGAGGAGACCG		
DN178487|c0_g3_i4	2	Fw	CGATGAGGCGGGACAATGTA	186	58
		Rw	TTCACCTGAGCACTCGACAC		
DN153617|c0_g2_i2	3	Fw	AGACCAGCCGACGAGACATA	120	56
		Rv	TCTCACCATCCCCGTCAAGT		
DN184913|c1_g2_i6	3	Fw	AGTGTCCACGAGCTTACTGC	178	58
		Rw	GGGCATTGCACAATACCGTC		
DN170202|c1_g1_i2	3	Fw	TGGCTGGACGTGTGGAATA	186	58
		Rw	GCAATGGCTAACAAGCAGGC		
DN148698|c0_g1_i1	3	Fw	CACAAAGGAGCGAGACGAGT	127	58
		Rw	TCAATGCCACACTGGGACAG		
DN108712|c1_g1_i1	3	Fw	TTCCACTGCCACAACCGAAT	122	56
		Rw	GCCTAACGCTCGATGTTCCT		
DN169625|c1_g2_i1	4	Fw	TCGACGGGAGGTCATCTTCA	159	58
		Rv	CCAGTGTTGGGAGACTCGTTT		
DN177453|c0_g1_i1	4	Fw	GCGACAGATACCGTTTTGCC	145	58
		Rw	TCAACTGTTCACCTGCCGTT		
DN113016|c0_g1_i1	4	Fw	GCGGAGTAGGTTCTCACCTG	148	60
		Rw	TGTGATGTGGAAAGGCGTCA		
DN136881|c0_g3_i1	4	Fw	TTGTGAACCCTCGCAAGACA	164	58
		Rv	CGGACATCCTGGCTTCAACA		

Melting curves were performed to detect nonspecific products and primer dimers and target specificity was confirmed by the presence of a single peak in each melt curve. Standard curves were prepared from serial dilutions of quantified amplicons. All PCR products were sequenced to confirm their identity. Control reactions were included in all runs to confirm the absence of genomic DNA. qPCR reaction efficiencies and r^2^ (coefficient of determination) were all > 90% for each target transcript. Expression normalization was performed using 18 S ribosomal RNA (18 S).

### Ethical approval

All applicable international, national and institutional guidelines for the care and use of animals were followed.

## Supplementary information

Supplementary Information.

Supplementary Information2.

Supplementary Information3.

Supplementary Information4.

Supplementary Information5.

Supplementary Information6.

Supplementary Information7.

## Data Availability

The sequence read data and sample information from this study were deposited in BioProject portal at NCBI and can be accessed through the following BioProject Accession no.: PRJNA532556. The datasets analysed in this study are publicly available and the sources are referenced in the text.
