# Supplementary material for: Holothurians have a reduced GPCR and odorant receptor-like repertoire compared to other echinoderms
Source: Sci Rep. 2020 Feb 25;10:3348. doi: 10.1038/s41598-020-60167-3 (PMC7042368; doi:10.1038/s41598-020-60167-3)
Supplement: Supplementary file 1 — Supplementary Information. [file 41598_2020_60167_MOESM1_ESM.pdf]

Holothurians have a reduced GPCR and odorant receptor-like repertoire compared to other echinoderms

Nathalie Marquet*, João C.R. Cardoso, Bruno Louro, Stefan A. Fernandes, Sandra C. Silva, Adelino V.M. Canário

CCMAR - Centre of Marine Sciences, University of Algarve, Campus de Gambelas, 8005-139 Faro, Portugal

*Corresponding author: N. Marquet

e-mail: <nmarquet@gmail.com>

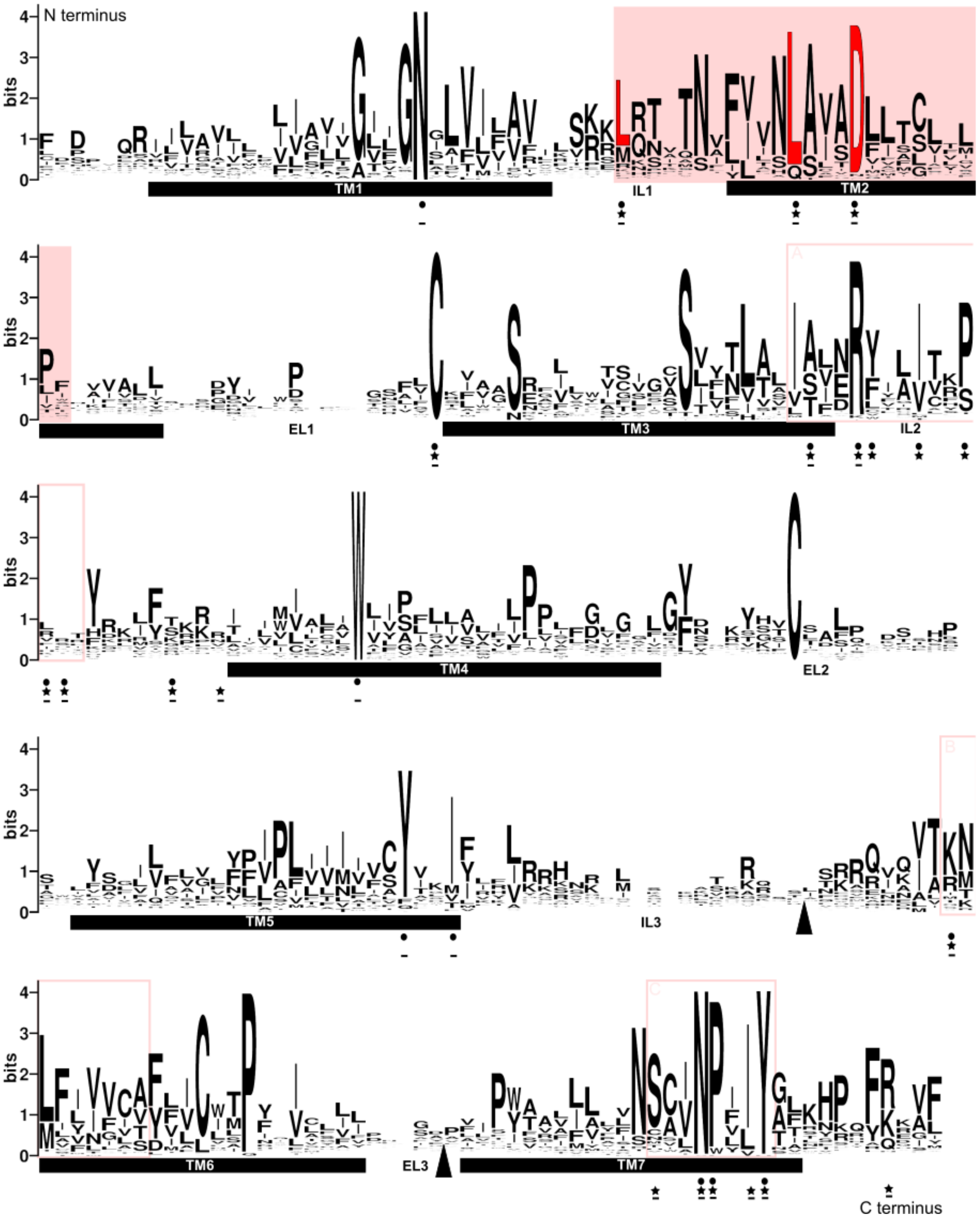

Supplementary Figure S1. WebLogo based on the alignment of the entire sea cucumber OR repertoire (*H. arguinensis* and *A. japonicus*). The black bars delimitate the seven transmembrane domains (TM1-7) defined in THMHMM v2.0 [72]. The intracellular and extracellular loops are labelled as "IL" and "EL" respectively. Positions with gaps in more than 95% sequences, poorly aligned blocks of sequences (arrows) and N- and C- terminal sequences have been removed. Amino acid positions that are common to fish ORs [84] are indicated with "●", and those common to cephalochordates [29] and cnidaria [28] are indicated with "★" and with "▬" respectively. The region of the alignment that contains the motif of interest is highlighted in pink. The pink frames represent the area where other motifs are usually found in ORs (A: MAxDRYxxxCxPLxY, B: KAxxTxxxH, C: PxxNPxxY). The height of each symbol indicates the relative frequency of each amino acid at that position.
