# Supplementary material for: Holothurians have a reduced GPCR and odorant receptor-like repertoire compared to other echinoderms
Source: Sci Rep. 2020 Feb 25;10:3348. doi: 10.1038/s41598-020-60167-3 (PMC7042368; doi:10.1038/s41598-020-60167-3)
Supplement: Supplementary file 2 — Supplementary Information2. [file 41598_2020_60167_MOESM2_ESM.pdf]

Holothurians have a reduced GPCR and odorant receptor-like repertoire compared to other echinoderms

Nathalie Marquet*, João C.R. Cardoso, Bruno Louro, Stefan A. Fernandes, Sandra C. Silva, Adelino V.M. Canário

CCMAR - Centre of Marine Sciences, University of Algarve, Campus de Gambelas, 8005-139 Faro, Portugal

*Corresponding author: N. Marquet

e-mail: <nmarquet@gmail.com>

Supplementary Note. Results of the Likelihood Ratio Tests.

Table 1. Results of the LRT based on branch-specific models (M0:M1).

Group	n	lnL0	lnL1	d.f.	LRT	P-value
1	44	-26591.4302	-26426.5890	84	329.6824	**<0.00E+00**
2	14	-8923.6870	-8903.5795	24	40.2150	2.03E-02
3	10	-11670.4460	-11646.3079	16	48.2763	**<4.30E-05**
4	14	-8378.7033	-8336.0917	24	85.2231	**<9.00E-09**

Table 2. Results of the LRT based on site specific models (M7:M8).

Group	n	lnL7	lnL8	d.f	LRT	P-value
1	44	-25904.6467	-25904.6664	2	0.039474	9.80E-01
2	14	-8739.5480	-8739.5507	2	0.005414	9.97E-01
3	10	-11365.8480	-11365.4573	2	0.781368	6.77E-01
4	14	-8195.8224	-8195.8273	2	0.009754	9.95E-01

LRT values were obtained with, 2 x (lnLb − lnLa), where a is the null hypothesis and b the alternative hypothesis. *P*-values in bold represent significant tests in which the *free-ratio* model (M1) was a better fit than the *one-ratio* model (M0). The *P*-values were calculated from the χ^2 distribution. *P*-values are Bonferroni corrected for the sequences in each group with α=0.05. n= Number of sequences in the group and d.f. = degree of freedom.

Supplementary information:

The amino acid sequences of two sea cucumber species identified in the four OR-l clades were aligned with CLUSTALW in MEGA 7 [1] using default settings. The PAL2NAL software [2] was then used to obtain multiple codon alignments based on the amino acid alignments and their corresponding nucleotide sequences. With those codon alignments, a phylogenetic tree for each group was created based on the Maximum-Likelihood method in MEGA 7 [1]. By estimating the codon substitution ratio (ω=dN/dS), one can infer the type of selective pressure acting on a set of sequences, where ω>1 indicates positive selection, ω=1 indicates neutral evolution and ω<1

indicates purifying selection [3]. With the likelihood ratio tests (LRT) calculated from the difference between the lnL values obtained from codon substitution models implemented in codeml (PAML 4.4 package), we aimed to test for variable selective pressures acting on four sea cucumber OR-clades. As suggested by Yang, et al. (2005) [4], the LRT were conducted to compare a null model that did not allow for $\omega > 1$ in the distribution with an alternative model that did. To do so, we applied two LRT tests. The first LRT was based on branch-specific models, comparing a free ratio model (M1) where the ω ratio of the branches in the phylogeny are free to vary, with a one ratio model (M0) where the ω value is fixed through all branches (Table 1). Since branch models average an ω value for the whole branch of a phylogeny, they have a limited power to identify positive selection that may only have acted on a few codons in the sequences [5]. We then proceeded to the second LRT based on site specific model, to identify selection pressure that may have acted on specific sites of the sequences (Table 2). Here, the LRT compared the beta model (M7) where the ω ratio is fixed in all the lineages of the phylogeny and implements ten classes of sites with $\omega \leq 1$ with the beta-ω (M8) which also implements a fixed omega for all the lineages of the phylogeny but attributes 11 classes of sites, 10 sites with $\omega \leq 1$, and one site with $\omega > 1$, allowing for positive selection. For each model, pairs the LRT statistic 2Δl, (twice the log likelihood difference) was compared with critical values retrieved from the χ^2 distribution as were their calculated *p*-value for which a Bonferroni correction for multiple testing was applied, as implemented by Yang (2007) [3].

References

1 Kumar, S., Stecher, G. & Tamura, K. MEGA7: Molecular Evolutionary Genetics Analysis Version 7.0 for Bigger Datasets. *Mol. Biol. Evol.* **33**, 1870-1874, doi:10.1093/molbev/msw054 (2016).

2 Suyama, M., Torrents, D. & Bork, P. PAL2NAL: robust conversion of protein sequence alignments into the corresponding codon alignments. *Nucleic Acids Res.* **34**, W609-612, doi:10.1093/nar/gkl315 (2006).

3 Yang, Z. PAML 4: phylogenetic analysis by maximum likelihood. *Mol. Biol. Evol.* **24**, 1586-1591, doi:10.1093/molbev/msm088 (2007).

4 Yang, Z., Wong, W. S. & Nielsen, R. Bayes empirical bayes inference of amino acid sites under positive selection. *Mol. Biol. Evol.* **22**, 1107-1118, doi:10.1093/molbev/msi097 (2005).

5 Anisimova, M., Bielawski, J. P. & Yang, Z. Accuracy and power of the likelihood ratio test in detecting adaptive molecular evolution. *Mol. Biol. Evol.* **18**, 1585-1592, doi:10.1093/oxfordjournals.molbev.a003945 (2001).
