# Supplementary material for: Holothurians have a reduced GPCR and odorant receptor-like repertoire compared to other echinoderms
Source: Sci Rep. 2020 Feb 25;10:3348. doi: 10.1038/s41598-020-60167-3 (PMC7042368; doi:10.1038/s41598-020-60167-3)
Supplement: Supplementary file 5 — Supplementary Information5. [file 41598_2020_60167_MOESM5_ESM.pdf]

Holothurians have a reduced GPCR and odorant receptor-like repertoire compared to other echinoderms

Nathalie Marquet*, João C.R. Cardoso, Bruno Louro, Stefan A. Fernandes, Sandra C. Silva, Adelino V.M. Canário

CCMAR - Centre of Marine Sciences, University of Algarve, Campus de Gambelas, 8005-139 Faro, Portugal

*Corresponding author: N. Marquet

e-mail: <nmarquet@gmail.com>

Supplementary Table S3. Results of the HMM analysis in *H. arguinensis*.

Protein ID	Hidden Markov Model (HMM)																	
	Sea urchin (Surreal)						Starfish											Sea anemone
	Group A	Group B	Group C	Group D	Group E	Group F	Group A	Group B	Group C	Group D	Group E	Group F	Group G	Group H	Group I	Group J	Group K	
TRINITY_DN101677_c0_g1_i1			1.8e-109											3.3e-59	7.4e-25	3.5e-13		
TRINITY_DN108528_c0_g1_i1			4.5e-61											1.2e-30	8,00E-57			
TRINITY_DN108712_c1_g1_i1				1.5e-169														
TRINITY_DN109505_c1_g1_i1			2.8e-86											1.3e-51	6.8e-31	1.3e-12		
TRINITY_DN113016_c0_g1_i1												2.1e-78						
TRINITY_DN114406_c0_g1_i1		1.2e-51								2.5e-91								
TRINITY_DN136692_c1_g1_i1			5.9e-25															
TRINITY_DN136881_c0_g3_i1												1.5e-56						
TRINITY_DN148436_c0_g1_i1		2.6e-49								7.9e-54								
TRINITY_DN148623_c0_g3_i1		4.0e-61								1.1e-66								
TRINITY_DN148698_c0_g1_i1				5.1e-62														
TRINITY_DN149306_c0_g2_i1												5.6e-90						
TRINITY_DN151046_c0_g1_i1			6.3e-144											8.1e-36	6.2e-20	4.9e-08		
TRINITY_DN151819_c0_g1_i1			2.0e-08															
TRINITY_DN152339_c0_g2_i1			2.0e-43											7.1e-07				
TRINITY_DN152517_c0_g2_i2			1.9e-26									3.3e-12						
TRINITY_DN153617_c0_g2_i2				9.7e-167														
TRINITY_DN154042_c0_g1_i1								7.0e-33	2.0e-28									
TRINITY_DN155888_c0_g1_i1			7.0e-10		3.4e-11			2.0e-10										
TRINITY_DN156703_c1_g1_i1			8.5e-32															
TRINITY_DN158222_c1_g1_i1			8.8e-13															
TRINITY_DN158856_c2_g2_i2		1.4e-46								1.8e-61								
TRINITY_DN159040_c0_g1_i1												1.1e-98						
TRINITY_DN160372_c0_g2_i1		8.5e-69								2.8e-63								
TRINITY_DN162010_c0_g1_i1		3.2e-63								3.1e-103								
TRINITY_DN164204_c0_g2_i1		3.9e-25								8.1e-86								
TRINITY_DN165100_c0_g1_i1		1.1e-97								3.3e-105								

TRINITY_DN165994_c1_g1_i1			3.3e-05		1.2e-06							7,00E-10						
TRINITY_DN165994_c1_g2_i1					1.3e-05							8,00E-08						
TRINITY_DN166152_c0_g4_i1								8.9e-13	3.8e-44									
TRINITY_DN167615_c0_g1_i3								8.8e-08										
TRINITY_DN168039_c0_g1_i2		3.9e-123								3.2e-166								
TRINITY_DN168321_c1_g3_i1		9.9e-61								1.9e-77								
TRINITY_DN168537_c1_g1_i1		3.3e-27								5.8e-86								
TRINITY_DN168797_c1_g4_i1			5.1e-86											8.2e-51	1.3e-36	1.2e-10		
TRINITY_DN169459_c1_g1_i1		4.6e-50								9.4e-82								
TRINITY_DN169527_c1_g2_i1		1.2e-51								6.7e-103								
TRINITY_DN169625_c1_g2_i1												2.5e-89						
TRINITY_DN170202_c1_g1_i2				2.1e-164														
TRINITY_DN170522_c0_g2_i1												1.6e-108						
TRINITY_DN170998_c0_g1_i1		4.5e-19								1.3e-58								
TRINITY_DN171647_c0_g1_i2		9.9e-108								2.9e-150								
TRINITY_DN172053_c0_g1_i1		5.2e-60								2.2e-99								
TRINITY_DN172574_c0_g1_i1																		
TRINITY_DN172862_c0_g1_i1												7.4e-98						
TRINITY_DN173209_c0_g1_i1		1.2e-107								1.5e-170								
TRINITY_DN173521_c0_g1_i1										8.6e-26								
TRINITY_DN173521_c0_g2_i1		1.1e-65								1.5e-91								
TRINITY_DN173654_c0_g1_i1		6.0e-106	4.3e-10							3.8e-168								
TRINITY_DN173682_c0_g3_i1									2.5e-11									
TRINITY_DN174721_c2_g1_i1		1.1e-109								8.8e-181								
TRINITY_DN174853_c0_g1_i1		2.4e-70								1.5e-88								
TRINITY_DN175107_c3_g1_i2		1.4e-66								2.8e-93								
TRINITY_DN176493_c0_g2_i1		8.2e-54								4.5e-60								
TRINITY_DN177453_c0_g1_i1												3.2e-62						
TRINITY_DN177751_c0_g4_i2			7.0e-36															
TRINITY_DN178050_c0_g1_i2					2.8e-18													
TRINITY_DN178387_c0_g1_i1								3.7e-10	7.3e-12									
TRINITY_DN178487_c0_g3_i4			4.1e-44															
TRINITY_DN178565_c0_g4_i1		1.8e-127	2.0e-07							6.9e-185								
TRINITY_DN178934_c0_g2_i7		1.5e-77								1.5e-134								
TRINITY_DN178934_c0_g6_i1										7.8e-58								
TRINITY_DN179851_c1_g5_i2		5.2e-89								2.1e-140								
TRINITY_DN181162_c0_g2_i1		3.0e-106								2.6e-131								
TRINITY_DN181254_c1_g3_i2			1.6e-53															
TRINITY_DN182323_c1_g7_i7		2.0e-88								7.8e-142								
TRINITY_DN184033_c1_g1_i1					2.6e-14			2.6e-09										1.1e-09
TRINITY_DN184550_c1_g1_i1		4.1e-35								2.5e-81								
TRINITY_DN184738_c2_g2_i1			4.5e-77											5.9e-51	9.5e-79			
TRINITY_DN184913_c1_g2_i6				6.5e-136														
TRINITY_DN186455_c3_g1_i1			4.3e-51															
TRINITY_DN186503_c2_g1_i1		1.6e-37								1.2e-27								
TRINITY_DN248782_c0_g1_i1			5.0e-96					2.4e-05						1.3e-21		1.4e-07		
TRINITY_DN61298_c0_g2_i1	2.5e-32																3.3e-22	
TRINITY_DN95037_c0_g2_i1		1.5e-44								8.9e-69								
TRINITY_DN96230_c1_g1_i1				3.6e-67														
TRINITY_DN97107_c0_g1_i1					1.3e-24			1.3e-05										
TRINITY_DN97579_c0_g1_i1			8.9e-83											1.6e-34		3.3e-15		

Supplementary Table S4. Results of the HMM analysis in *A. japonicus*.

	Hidden Markov Model (HMM)																	
Protein ID	Sea urchin (Surreal)						Starfish											Sea anemone
	Group A	Group B	Group C	Group D	Group E	Group F	Group A	Group B	Group C	Group D	Group E	Group F	Group G	Group H	Group I	Group J	Group K	
Aja.PIK33197.1								5.5e-11	3.3e-13									
Aja.PIK33210.1		3.2e-18								7.7e-77								
Aja.PIK33580.1		1.3e-24								2.5e-68								
Aja.PIK33772.1		4.9e-84								1.8e-101								
Aja.PIK33908.1			1.7e-06															
Aja.PIK34350.1										9.7e-50								
Aja.PIK34351.1										5.2e-51								
Aja.PIK34355.1		2.1e-61								5.3e-87								
Aja.PIK34888.1			7.1e-65											7.0e-41	1.0e-17			
Aja.PIK35204.1												6.9e-59						
Aja.PIK35427.1		4.5e-29								6.9e-59								
Aja.PIK35879.1			2.0e-98											2.4e-64	5.1e-38	4.6e-09		
Aja.PIK36567.1										1.6e-37								
Aja.PIK36688.1		4.9e-23								1.8e-68								
Aja.PIK36693.1		5.1e-121								1.0e-115								
Aja.PIK36694.1		1.3e-24								9.1e-36								
Aja.PIK37406.1												8.5e-58						
Aja.PIK37689.1								1.9e-09										
Aja.PIK37947.1				1.5e-81														
Aja.PIK38193.1			1.6e-114											7.7e-28	5.3e-08	8.5e-10		
Aja.PIK39029.1												2.8e-76						
Aja.PIK39287.1								2.1e-24	7.6e-28									
Aja.PIK39302.1				5.1e-166														
Aja.PIK39336.1			1.2e-76											4.4e-48	4,00E-76	2.6e-15		
Aja.PIK39864.1										6.9e-22								
Aja.PIK39897.1			1.7e-40											3.7e-27				
Aja.PIK39927.1				5.1e-147														
Aja.PIK40478.1						1.2e-78							1.4e-12					
Aja.PIK41151.1								2.8e-10	2.4e-11									
Aja.PIK41271.1				2.1e-171														
Aja.PIK41413.1		3.1e-78								6.2e-100								
Aja.PIK41414.1		7.3e-40								1.7e-56								
Aja.PIK41415.1		5.9e-74								1.2e-97								
Aja.PIK41591.1										4.2e-51								
Aja.PIK41601.1										8.1e-26								
Aja.PIK42805.1			1.2e-12		1.8e-19			1.7e-16	1.2e-07									
Aja.PIK42823.1			7.8e-12		1.1e-15			6.1e-16	8.8e-10									
Aja.PIK43000.1			1.5e-38															
Aja.PIK43106.1					7.8e-15													
Aja.PIK43110.1					2.8e-19													
Aja.PIK43514.1					6.8e-14			4.3e-05										
Aja.PIK43705.1			4.8e-06															
Aja.PIK43739.1			4.6e-99											3.4e-34	2.3e-58			

Aja.PIK44163.1										4.5e-21								
Aja.PIK44261.1										1.1e-24								
Aja.PIK44608.1		4.9e-23								1.8e-68								
Aja.PIK45041.1		4.4e-54								3.9e-43								
Aja.PIK45612.1		6.4e-74								5.7e-74								
Aja.PIK45659.1			6.3e-82											1.2e-55	1.0e-40	1.2e-14		
Aja.PIK46163.1			3.2e-61											8.6e-41	1.2e-06			
Aja.PIK46164.1										2.0e-15								
Aja.PIK46165.1										5.3e-21								
Aja.PIK46220.1			1.7e-89											2.2e-46	1.5e-21	8.7e-11		
Aja.PIK46422.1		6.4e-59								5.4e-99								
Aja.PIK46423.1										6.5e-54								
Aja.PIK46424.1										1.7e-49								
Aja.PIK47942.1		1.6e-57								7.0e-124								
Aja.PIK48119.1																		
Aja.PIK48217.1																		
Aja.PIK48327.1				9.8e-207														
Aja.PIK48567.1			5.7e-95											2.0e-58	6.4e-106	8.6e-12		
Aja.PIK48719.1												2.0e-15						
Aja.PIK49159.1		1.7e-32								4.0e-60								
Aja.PIK49275.1			3.8e-19		3.4e-19			9.4e-10										4.8e-8
Aja.PIK49674.1		8.1e-86								2.5e-77								
Aja.PIK49846.1		2.9e-114	1.5e-06							2.4e-170								
Aja.PIK49850.1												1.7e-82						
Aja.PIK50125.1			2.2e-42															
Aja.PIK50265.1												1.5e-67						
Aja.PIK51166.1			2.4e-105											3.9e-53	5.8e-18	2.3e-100		
Aja.PIK51242.1			1.1e-42															
Aja.PIK51243.1			6.5e-78											1.9e-06				
Aja.PIK51246.1			4.8e-46															
Aja.PIK51747.1			3.3e-36															
Aja.PIK51748.1			4.7e-85											8.8e-59	3.8e-25			
Aja.PIK51887.1		2.5e-86								1.3e-108								
Aja.PIK52122.1				2.6e-95														
Aja.PIK52319.1				5.2e-67														
Aja.PIK52842.1																		
Aja.PIK53409.1		6.8e-43								1.5e-77								
Aja.PIK53505.1																		
Aja.PIK53655.1																		
Aja.PIK53684.1										3.0e-18								
Aja.PIK53869.1		1.2e-22								3.9e-84								
Aja.PIK53870.1		3.9e-89								2.6e-138								
Aja.PIK53952.1									2.7e-16									
Aja.PIK54287.1												2.9e-51						
Aja.PIK54619.1		5.1e-109								6,00E-157								
Aja.PIK54620.1		7.3e-84								4.9e-145								
Aja.PIK54803.1			2.3e-113											1.8e-59	6.4e-31	4.9e-11		
Aja.PIK55050.1												4.7e-100						
Aja.PIK55199.1												2.0e-110						
Aja.PIK55588.1			1.3e-45															
Aja.PIK55903.1			4.4e-11															

Aja.PIK55943.1																		
Aja.PIK55946.1		9.8e-110			3.6e-6					8.3e-118								
Aja.PIK55947.1		3.4e-109			6.7e-7					5.4e-115								
Aja.PIK56143.1	1.6e-31					1.7e-7											1.2e-25	
Aja.PIK56235.1										3.3e-58								
Aja.PIK56272.1						1.7e-30												
Aja.PIK56697.1		8.4e-62								1.2e-97								
Aja.PIK58353.1		1.6e-56								2.9e-63								
Aja.PIK58426.1																		
Aja.PIK58680.1					3.6e-11													
Aja.PIK58689.1			9.9e-07		9.0e-27			6.1e-16	2.2e-05									5.9e-16
Aja.PIK58690.1					9.4e-21													8.1e-7
Aja.PIK58691.1					2.7e-20			1.7e-05										
Aja.PIK59092.1				4.3e-155														
Aja.PIK61132.1			1.4e-83											2.2e-44	1.9e-31			
Aja.PIK61414.1		1.7e-32								6.2e-67								
Aja.PIK61481.1			6.3e-14		9.0e-23													
Aja.PIK62784.1					3.4e-12													
