# Supplementary material for: Holothurians have a reduced GPCR and odorant receptor-like repertoire compared to other echinoderms
Source: Sci Rep. 2020 Feb 25;10:3348. doi: 10.1038/s41598-020-60167-3 (PMC7042368; doi:10.1038/s41598-020-60167-3)
Supplement: Supplementary file 7 — Supplementary Information7. [file 41598_2020_60167_MOESM7_ESM.pdf]

Holothurians have a reduced GPCR and odorant receptor-like repertoire compared to other echinoderms

Nathalie Marquet*, João C.R. Cardoso, Bruno Louro, Stefan A. Fernandes, Sandra C. Silva, Adelino V.M. Canário

CCMAR - Centre of Marine Sciences, University of Algarve, Campus de Gambelas, 8005-139 Faro, Portugal

*Corresponding author: N. Marquet

e-mail: <nmarquet@gmail.com>

Supplementary Table S6. Results of the tBLASTn of the OR-like candidates in *H. arguinensis* against the six tissue assemblies.

Tissue	HMM file	Individual assembly	Identity	Coverage	Score	Bits	e value
Calcareous R	TRINITY_DN174721_c2_g1_i1	TR62289|c0_g1_i1	100.000	154	2	463	7.88e-110
Calcareous R	TRINITY_DN181162_c0_g2_i1	TR113471|c0_g1_i3	100.000	156	2	469	1.18e-108
Calcareous R	TRINITY_DN168039_c0_g1_i2	TR108995|c0_g1_i1	100.000	162	487	2	1.37e-87
Calcareous R	TRINITY_DN173209_c0_g1_i1	TR108086|c0_g1_i1	100.000	164	2	493	2.23e-115
Calcareous R	TRINITY_DN184738_c2_g2_i1	TR86425|c0_g2_i1	100.000	166	500	3	2.09e-121
Calcareous R	TRINITY_DN184738_c2_g2_i1	TR86425|c0_g1_i1	100.000	166	500	3	2.09e-121
Calcareous R	TRINITY_DN164204_c0_g2_i1	TR71936|c0_g1_i1	100.000	167	1	501	2.52e-117
Calcareous R	TRINITY_DN170202_c1_g1_i2	TR37534|c0_g1_i1	100.000	168	506	3	1.50e-101
Calcareous R	TRINITY_DN170522_c0_g2_i1	TR67961|c0_g1_i1	100.000	203	1	609	7.53e-138
Calcareous R	TRINITY_DN168797_c1_g4_i1	TR101818|c0_g1_i1	100.000	206	620	3	4.73e-135
Calcareous R	TRINITY_DN174853_c0_g1_i1	TR56591|c0_g1_i1	100.000	230	1	690	8.43e-141
Calcareous R	TRINITY_DN186503_c2_g1_i1	TR112218|c1_g1_i1	100.000	260	780	1	0.0
Calcareous R	TRINITY_DN179851_c1_g5_i2	TR99143|c0_g1_i2	100.000	264	1	792	2.71e-178
Calcareous R	TRINITY_DN179851_c1_g5_i2	TR99143|c0_g1_i2	100.000	264	2107	1316	2.71e-178
Calcareous R	TRINITY_DN178565_c0_g4_i1	TR59540|c0_g1_i1	100.000	292	878	3	0.0
Calcareous R	TRINITY_DN169625_c1_g2_i1	TR66420|c0_g3_i1	97.115	208	2	625	7.85e-149
Calcareous R	TRINITY_DN184913_c1_g2_i6	TR109287|c1_g1_i1	97.525	202	606	1	3.27e-124
Calcareous R	TRINITY_DN168321_c1_g3_i1	TR109175|c0_g1_i1	97.605	334	1	1002	0.0
Calcareous R	TRINITY_DN184550_c1_g1_i1	TR75332|c0_g1_i1	97.674	344	1982	951	0.0
Calcareous R	TRINITY_DN184550_c1_g1_i1	TR75332|c0_g1_i3	97.971	345	2013	979	0.0
Calcareous R	TRINITY_DN178934_c0_g2_i7	TR80371|c0_g2_i1	98.380	432	147	1439	0.0
Calcareous R	TRINITY_DN181162_c0_g2_i1	TR113471|c0_g1_i2	98.690	229	2	688	9.73e-142
Calcareous R	TRINITY_DN181162_c0_g2_i1	TR113471|c0_g1_i1	98.690	229	2	688	4.35e-140
Calcareous R	TRINITY_DN162010_c0_g1_i1	TR95673|c0_g2_i1	99.045	314	1018	77	0.0

Calcareous R	TRINITY_DN175107_c3_g1_i2	TR47144|c0_g1_i1	99.134	231	695	3	1.80e-133
Calcareous R	TRINITY_DN173654_c0_g1_i1	TR3430|c0_g1_i1	99.338	151	589	137	8.71e-86
Oral cavity	TRINITY_DN148436_c0_g1_i1	TR97282|c0_g1_i1	100.000	153	459	1	1.38e-111
Oral cavity	TRINITY_DN159040_c0_g1_i1	TR129728|c0_g1_i2	100.000	154	42	503	2.16e-107
Oral cavity	TRINITY_DN148698_c0_g1_i1	TR89378|c0_g2_i1	100.000	175	527	3	1.18e-100
Oral cavity	TRINITY_DN169527_c1_g2_i1	TR64194|c0_g2_i2	100.000	179	1152	616	1.39e-127
Oral cavity	TRINITY_DN169527_c1_g2_i1	TR64194|c0_g2_i1	100.000	179	1398	862	2.03e-126
Oral cavity	TRINITY_DN108712_c1_g1_i1	TR118155|c0_g1_i1	100.000	196	588	1	9.04e-128
Oral cavity	TRINITY_DN178487_c0_g3_i4	TR71822|c0_g1_i1	100.000	207	858	238	4.98e-150
Oral cavity	TRINITY_DN109505_c1_g1_i1	TR78393|c0_g1_i1	100.000	219	2	658	2.91e-149
Oral cavity	TRINITY_DN149306_c0_g2_i1	TR74832|c0_g1_i1	100.000	225	676	2	7.16e-163
Oral cavity	TRINITY_DN160372_c0_g2_i1	TR60316|c0_g2_i1	100.000	246	738	1	1.19e-151
Oral cavity	TRINITY_DN169625_c1_g2_i1	TR82014|c0_g1_i1	100.000	301	1412	510	0.0
Oral cavity	TRINITY_DN186503_c2_g1_i1	TR58919|c2_g1_i2	100.000	307	1595	675	0.0
Oral cavity	TRINITY_DN177453_c0_g1_i1	TR80668|c0_g1_i1	100.000	317	2	952	0.0
Oral cavity	TRINITY_DN174853_c0_g1_i1	TR86962|c0_g1_i1	100.000	345	285	1319	0.0
Oral cavity	TRINITY_DN179851_c1_g5_i2	TR88782|c1_g1_i4	100.000	401	795	1997	0.0
Oral cavity	TRINITY_DN184913_c1_g2_i6	TR102092|c0_g2_i9	97.006	835	3363	862	0.0
Oral cavity	TRINITY_DN184550_c1_g1_i1	TR59887|c1_g2_i6	97.093	344	12	1043	0.0
Oral cavity	TRINITY_DN174721_c2_g1_i1	TR129837|c1_g3_i4	97.333	150	452	3	2.17e-84
Oral cavity	TRINITY_DN184913_c1_g2_i6	TR102092|c0_g2_i1	97.445	274	1683	862	0.0
Oral cavity	TRINITY_DN184550_c1_g1_i1	TR59887|c1_g2_i3	97.674	344	12	1043	0.0
Oral cavity	TRINITY_DN169527_c1_g2_i1	TR64194|c0_g1_i1	97.714	175	526	2	5.20e-124
Oral cavity	TRINITY_DN168321_c1_g3_i1	TR92371|c1_g1_i4	97.865	281	1	843	0.0
Oral cavity	TRINITY_DN168321_c1_g3_i1	TR92371|c1_g1_i3	97.865	281	1	843	0.0
Oral cavity	TRINITY_DN184913_c1_g2_i6	TR102092|c0_g2_i7	97.964	835	3261	757	0.0
Oral cavity	TRINITY_DN152339_c0_g2_i1	TR49255|c0_g1_i1	98.077	208	623	3	4.26e-129
Oral cavity	TRINITY_DN184913_c1_g2_i6	TR102092|c0_g2_i3	98.204	835	3367	863	0.0
Oral cavity	TRINITY_DN184738_c2_g2_i1	TR60438|c0_g1_i1	98.235	170	2	511	6.62e-117
Oral cavity	TRINITY_DN165100_c0_g1_i1	TR93990|c0_g1_i1	98.408	377	1822	692	0.0
Oral cavity	TRINITY_DN168537_c1_g1_i1	TR94296|c0_g1_i1	98.462	325	36	1010	0.0
Oral cavity	TRINITY_DN170998_c0_g1_i1	TR93811|c0_g1_i2	98.512	336	1	1008	0.0
Oral cavity	TRINITY_DN175107_c3_g1_i2	TR121109|c0_g1_i1	98.812	505	1	1515	0.0
Oral cavity	TRINITY_DN178934_c0_g2_i7	TR123470|c2_g1_i3	98.820	339	53	1066	0.0
Oral cavity	TRINITY_DN184550_c1_g1_i1	TR59887|c1_g2_i1	98.837	344	12	1043	0.0
Oral cavity	TRINITY_DN176493_c0_g2_i1	TR100585|c2_g1_i1	98.944	284	891	40	1.96e-175
Oral cavity	TRINITY_DN148698_c0_g1_i1	TR89378|c0_g1_i1	98.983	295	1197	316	8.72e-174
Oral cavity	TRINITY_DN169459_c1_g1_i1	TR127632|c0_g1_i1	99.020	204	1569	958	3.46e-133
Oral cavity	TRINITY_DN164204_c0_g2_i1	TR128998|c0_g1_i1	99.065	321	3	965	0.0
Oral cavity	TRINITY_DN168797_c1_g4_i1	TR65225|c2_g1_i1	99.070	215	2	646	3.21e-137
Oral cavity	TRINITY_DN182323_c1_g7_i7	TR77581|c1_g1_i1	99.167	240	2424	1705	4.29e-153
Oral cavity	TRINITY_DN178934_c0_g2_i7	TR123470|c2_g1_i4	99.306	432	377	1669	0.0
Oral cavity	TRINITY_DN178934_c0_g2_i7	TR123470|c2_g1_i1	99.306	432	377	1669	0.0
Oral cavity	TRINITY_DN162010_c0_g1_i1	TR78372|c1_g1_i1	99.373	319	3	959	0.0
Oral cavity	TRINITY_DN186503_c2_g1_i1	TR58919|c1_g1_i1	99.405	168	145	648	1.65e-118
Oral cavity	TRINITY_DN175107_c3_g1_i2	TR121109|c0_g1_i2	99.406	505	1	1515	0.0

Oral cavity	TRINITY_DN168797_c1_g4_i1	TR107252|c0_g1_i1	99.415	171	99	611	8.92e-118
Oral cavity	TRINITY_DN113016_c0_g1_i1	TR12197|c0_g1_i1	99.487	195	695	111	3.85e-142
Oral cavity	TRINITY_DN179851_c1_g5_i2	TR88782|c1_g1_i5	99.501	401	792	1994	0.0
Oral cavity	TRINITY_DN179851_c1_g5_i2	TR88782|c1_g1_i1	99.501	401	794	1996	0.0
Oral cavity	TRINITY_DN178934_c0_g2_i7	TR123470|c2_g1_i2	99.537	432	377	1669	0.0
Oral cavity	TRINITY_DN171647_c0_g1_i2	TR129624|c0_g1_i1	99.558	226	2	679	1.81e-122
Oral cavity	TRINITY_DN174721_c2_g1_i1	TR129837|c1_g3_i2	99.595	247	908	168	2.82e-163
Oral cavity	TRINITY_DN174721_c2_g1_i1	TR129837|c1_g3_i3	99.595	247	926	186	3.50e-163
Oral cavity	TRINITY_DN114406_c0_g1_i1	TR125911|c0_g1_i1	99.634	273	1	819	0.0
Oral cavity	TRINITY_DN178565_c0_g4_i1	TR53291|c1_g1_i1	99.650	286	1	858	0.0
Oral cavity	TRINITY_DN173209_c0_g1_i1	TR67244|c0_g1_i1	99.677	310	1556	627	0.0
Oral cavity	TRINITY_DN170202_c1_g1_i2	TR81166|c0_g1_i1	99.687	319	1186	230	0.0
Oral cavity	TRINITY_DN181162_c0_g2_i1	TR69298|c0_g1_i5	99.714	350	1536	487	0.0
Oral cavity	TRINITY_DN181162_c0_g2_i1	TR69298|c0_g1_i2	99.714	350	1745	696	0.0
Oral cavity	TRINITY_DN181162_c0_g2_i1	TR69298|c0_g1_i1	99.714	350	1865	816	0.0
Oral cavity	TRINITY_DN168039_c0_g1_i2	TR104640|c0_g1_i1	99.723	361	1259	177	0.0
Oral cavity	TRINITY_DN173654_c0_g1_i1	TR70268|c0_g1_i1	99.736	379	1696	560	0.0
Oral cavity	TRINITY_DN179851_c1_g5_i2	TR88782|c1_g1_i3	99.751	401	795	1997	0.0
Oral cavity	TRINITY_DN179851_c1_g5_i2	TR88782|c1_g1_i2	99.751	401	795	1997	0.0
Oral cavity	TRINITY_DN179851_c1_g5_i2	TR88782|c1_g1_i6	99.751	401	794	1996	0.0
Oral cavity	TRINITY_DN178934_c0_g2_i7	TR123470|c2_g1_i5	99.769	432	377	1669	0.0
Ovary	TRINITY_DN173654_c0_g1_i1	TR24297|c0_g1_i1	100.000	202	1	606	9.74e-137
Ovary	TRINITY_DN178565_c0_g4_i1	TR26306|c0_g1_i1	100.000	292	3	878	0.0
Ovary	TRINITY_DN182323_c1_g7_i7	TR21382|c0_g1_i1	98.913	184	2	553	1.32e-119
Ovary	TRINITY_DN179851_c1_g5_i2	TR40164|c0_g1_i1	99.444	180	1	540	4.84e-119
Ovary	TRINITY_DN182323_c1_g7_i7	TR21382|c1_g1_i1	99.454	183	258	806	1.17e-128
Papillae	TRINITY_DN177453_c0_g1_i1	TR41363|c0_g1_i1	100.000	152	3	458	2.93e-101
Papillae	TRINITY_DN169527_c1_g2_i1	TR108417|c0_g1_i1	100.000	157	3	473	5.74e-114
Papillae	TRINITY_DN173209_c0_g1_i1	TR93069|c0_g1_i1	100.000	159	479	3	9.22e-112
Papillae	TRINITY_DN159040_c0_g1_i1	TR126304|c0_g1_i1	100.000	165	1	495	3.38e-101
Papillae	TRINITY_DN177751_c0_g4_i2	TR108267|c0_g1_i1	100.000	171	1985	1473	9.48e-106
Papillae	TRINITY_DN148698_c0_g1_i1	TR24191|c0_g1_i1	100.000	180	3	542	7.35e-109
Papillae	TRINITY_DN173654_c0_g1_i1	TR109277|c0_g2_i1	100.000	183	853	305	3.93e-109
Papillae	TRINITY_DN136881_c0_g3_i1	TR59882|c0_g1_i1	100.000	208	2	625	1.33e-152
Papillae	TRINITY_DN170202_c1_g1_i2	TR94283|c1_g1_i1	100.000	208	719	96	2.23e-140
Papillae	TRINITY_DN173654_c0_g1_i1	TR109277|c0_g1_i1	100.000	234	704	3	1.10e-143
Papillae	TRINITY_DN184738_c2_g2_i1	TR68502|c0_g1_i1	100.000	254	970	209	1.55e-179
Papillae	TRINITY_DN174853_c0_g1_i1	TR82784|c0_g2_i1	100.000	364	186	1277	0.0
Papillae	TRINITY_DN174721_c2_g1_i1	TR106511|c0_g1_i1	97.384	344	1057	26	0.0
Papillae	TRINITY_DN178487_c0_g3_i4	TR105454|c0_g2_i1	97.727	264	353	1144	4.91e-164
Papillae	TRINITY_DN184913_c1_g2_i6	TR8363|c1_g1_i9	97.983	843	39	2564	0.0
Papillae	TRINITY_DN184913_c1_g2_i6	TR8363|c1_g1_i8	97.983	843	39	2564	0.0
Papillae	TRINITY_DN184913_c1_g2_i6	TR8363|c1_g1_i7	97.983	843	39	2564	0.0
Papillae	TRINITY_DN184913_c1_g2_i6	TR8363|c1_g1_i6	97.983	843	39	2564	0.0
Papillae	TRINITY_DN184913_c1_g2_i6	TR8363|c1_g1_i5	97.983	843	39	2564	0.0
Papillae	TRINITY_DN184913_c1_g2_i6	TR8363|c1_g1_i3	97.983	843	39	2564	0.0

Papillae	TRINITY_DN184913_c1_g2_i6	TR8363|c1_g1_i2	97.983	843	39	2564	0.0
Papillae	TRINITY_DN184913_c1_g2_i6	TR8363|c1_g1_i1	97.983	843	39	2564	0.0
Papillae	TRINITY_DN175107_c3_g1_i2	TR23135|c2_g1_i3	98.026	304	3	914	0.0
Papillae	TRINITY_DN184913_c1_g2_i6	TR8363|c1_g1_i10	98.102	843	39	2564	0.0
Papillae	TRINITY_DN184913_c1_g2_i6	TR8363|c1_g1_i4	98.102	843	39	2564	0.0
Papillae	TRINITY_DN168537_c1_g1_i1	TR110512|c0_g2_i1	98.107	317	1055	105	0.0
Papillae	TRINITY_DN168537_c1_g1_i1	TR110512|c0_g2_i2	98.125	320	1327	368	0.0
Papillae	TRINITY_DN181162_c0_g2_i1	TR501|c0_g1_i2	98.286	350	1438	389	0.0
Papillae	TRINITY_DN160372_c0_g2_i1	TR84865|c0_g1_i1	98.500	200	23	622	1.55e-132
Papillae	TRINITY_DN168797_c1_g4_i1	TR78281|c0_g1_i2	98.556	277	1511	681	0.0
Papillae	TRINITY_DN181162_c0_g2_i1	TR501|c0_g1_i5	98.571	350	1438	389	0.0
Papillae	TRINITY_DN160372_c0_g2_i1	TR126053|c0_g1_i1	98.605	215	658	14	1.12e-154
Papillae	TRINITY_DN179851_c1_g5_i2	TR36986|c0_g2_i3	98.635	293	795	1664	0.0
Papillae	TRINITY_DN175107_c3_g1_i2	TR23135|c2_g1_i1	98.654	520	3	1562	0.0
Papillae	TRINITY_DN181162_c0_g2_i1	TR501|c0_g1_i1	98.658	298	1075	182	0.0
Papillae	TRINITY_DN181162_c0_g2_i1	TR501|c0_g1_i3	98.857	350	1238	189	0.0
Papillae	TRINITY_DN153617_c0_g2_i2	TR112560|c0_g1_i1	98.953	191	6	578	1.22e-133
Papillae	TRINITY_DN178565_c0_g4_i1	TR34341|c0_g4_i2	98.974	390	708	1877	0.0
Papillae	TRINITY_DN172862_c0_g1_i1	TR88895|c0_g2_i1	98.987	395	1289	105	0.0
Papillae	TRINITY_DN152339_c0_g2_i1	TR127203|c0_g1_i1	99.013	304	910	2	0.0
Papillae	TRINITY_DN176493_c0_g2_i1	TR50777|c2_g3_i1	99.034	207	623	3	8.33e-127
Papillae	TRINITY_DN168321_c1_g3_i1	TR56790|c0_g3_i1	99.057	318	1541	588	0.0
Papillae	TRINITY_DN182323_c1_g7_i7	TR112065|c0_g1_i2	99.065	321	61	1023	0.0
Papillae	TRINITY_DN184550_c1_g1_i1	TR76814|c0_g1_i1	99.138	232	696	1	4.22e-134
Papillae	TRINITY_DN182323_c1_g7_i7	TR112065|c0_g1_i1	99.145	351	61	1113	0.0
Papillae	TRINITY_DN162010_c0_g1_i1	TR125382|c0_g1_i1	99.174	242	976	251	3.88e-160
Papillae	TRINITY_DN164204_c0_g2_i1	TR72362|c1_g1_i1	99.180	244	1165	434	8.47e-175
Papillae	TRINITY_DN179851_c1_g5_i2	TR36986|c0_g2_i9	99.252	401	793	1995	0.0
Papillae	TRINITY_DN186503_c2_g1_i1	TR45189|c3_g1_i1	99.389	491	2206	734	0.0
Papillae	TRINITY_DN179851_c1_g5_i2	TR36986|c0_g2_i8	99.413	341	675	1697	0.0
Papillae	TRINITY_DN175107_c3_g1_i2	TR23135|c2_g1_i2	99.423	520	3	1562	0.0
Papillae	TRINITY_DN181162_c0_g2_i1	TR501|c0_g1_i4	99.429	350	1481	432	0.0
Papillae	TRINITY_DN181162_c0_g2_i1	TR501|c0_g1_i7	99.429	350	1238	189	0.0
Papillae	TRINITY_DN181162_c0_g2_i1	TR501|c0_g1_i6	99.429	350	1481	432	0.0
Papillae	TRINITY_DN171647_c0_g1_i2	TR92951|c1_g1_i2	99.556	225	1541	867	3.21e-122
Papillae	TRINITY_DN169625_c1_g2_i1	TR21167|c2_g2_i1	99.659	293	1379	501	0.0
Papillae	TRINITY_DN170998_c0_g1_i1	TR112700|c0_g2_i2	99.731	372	1541	426	0.0
Papillae	TRINITY_DN170998_c0_g1_i1	TR112700|c0_g2_i1	99.731	372	1558	443	0.0
Papillae	TRINITY_DN178565_c0_g4_i1	TR34341|c0_g4_i1	99.741	386	708	1865	0.0
Papillae	TRINITY_DN179851_c1_g5_i2	TR36986|c0_g2_i7	99.751	401	793	1995	0.0
Papillae	TRINITY_DN179851_c1_g5_i2	TR36986|c0_g2_i2	99.751	401	794	1996	0.0
Tentacles	TRINITY_DN155888_c0_g1_i1	TR33953|c0_g1_i1	100.000	172	3	518	6.14e-102
Tentacles	TRINITY_DN168039_c0_g1_i2	TR39154|c0_g1_i1	100.000	176	11	538	1.86e-128
Tentacles	TRINITY_DN151046_c0_g1_i1	TR59336|c0_g1_i1	100.000	228	2	685	3.22e-163
Tentacles	TRINITY_DN178565_c0_g4_i1	TR68730|c0_g1_i1	100.000	253	1	759	1.94e-169
Tentacles	TRINITY_DN108528_c0_g1_i1	TR72173|c0_g1_i1	100.000	280	3	842	0.0

Tentacles	TRINITY_DN184738_c2_g2_i1	TR66322|c0_g1_i1	100.000	330	209	1198	0.0
Tentacles	TRINITY_DN177453_c0_g1_i1	TR63053|c1_g1_i1	100.000	386	23	1180	0.0
Tentacles	TRINITY_DN175107_c3_g1_i2	TR95048|c1_g1_i1	98.225	169	507	1	1.27e-110
Tentacles	TRINITY_DN174853_c0_g1_i1	TR78994|c0_g1_i1	98.817	169	713	207	2.31e-92
Tentacles	TRINITY_DN174853_c0_g1_i1	TR97800|c0_g2_i1	99.383	162	225	710	1.21e-111
Tentacles	TRINITY_DN174853_c0_g1_i1	TR97800|c0_g1_i1	99.383	162	226	711	1.26e-111
Tentacles	TRINITY_DN166152_c0_g4_i1	TR72715|c0_g1_i1	99.383	162	487	2	2.05e-115
Tentacles	TRINITY_DN113016_c0_g1_i1	TR72867|c1_g1_i1	99.408	169	507	1	4.41e-106
Tentacles	TRINITY_DN151819_c0_g1_i1	TR22654|c0_g1_i1	99.429	175	526	2	1.78e-112
Tentacles	TRINITY_DN182323_c1_g7_i7	TR58983|c0_g1_i1	99.636	275	825	1	0.0
Tentacles	TRINITY_DN175107_c3_g1_i2	TR95048|c2_g1_i1	99.642	279	1142	306	1.40e-166
Tentacles	TRINITY_DN178934_c0_g2_i7	TR61985|c0_g1_i1	99.660	294	880	2	0.0
Tentacles	TRINITY_DN159040_c0_g1_i1	TR61687|c0_g1_i1	99.717	353	1059	1	0.0
